# Distributive Nd-to-Yb Energy Transfer within Pure [YbNdYb] Heterometallic Molecules

**DOI:** 10.1021/acs.inorgchem.2c03940

**Published:** 2023-02-08

**Authors:** Diamantoula Maniaki, Annika Sickinger, Leoní A. Barrios Moreno, David Aguilà, Olivier Roubeau, Nicholas S. Settineri, Yannick Guyot, François Riobé, Olivier Maury, Laura Abad Galán, Guillem Aromí

**Affiliations:** † Departament de Química Inorgànica i Orgànica, Universitat de Barcelona, Diagonal 645, 08028 Barcelona, Spain; ‡ Institute of Nanoscience and Nanotechnology of the University of Barcelona (IN2UB), 08028 Barcelona, Spain; § Departamento de Química Inorgánica, Universidad Complutense de Madrid, Avda. Complutense s/n, 28040 Madrid, Spain; ∥ Instituto de Nanociencia y Materiales de Aragón (INMA), CSIC and Universidad de Zaragoza, Plaza San Francisco s/n, 50009 Zaragoza, Spain; ⊥ Advanced Light Source, Berkeley Laboratory, 1 Cyclotron Road, Berkeley, California 94720, United States; # Department of Chemistry, University of California, Berkeley, Berkeley, California 94720, United States; ∇ Laboratoire de Chimie, UMR 5182, CNRS, ENS Lyon, Univ Lyon, F69342 Lyon, France; ○ Institut Lumière Matière, UMR 5306 CNRS—Université Claude Bernard, Univ. Lyon, Lyon 1, 10 rue Ada Byron, F-69622 Villeurbanne Cedex, France

## Abstract

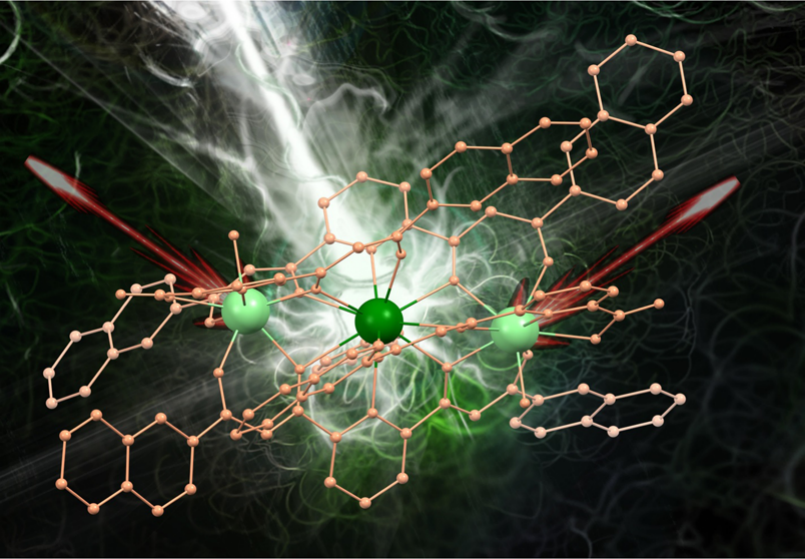

Facile access to site-selective hetero-lanthanide molecules will open new avenues in the search of novel photophysical phenomena based on Ln-to-Ln′ energy transfer (ET). This challenge demands strategies to segregate efficiently different Ln metal ions among different positions in a molecule. We report here the one-step synthesis and structure of a pure [YbNdYb] (**1**) coordination complex featuring short Yb···Nd distances, ideal to investigate a potential distributive (*i.e.*, from one donor to two acceptors) intramolecular ET from one Nd^3+^ ion to two Yb^3+^ centers within a well-characterized molecule. The difference in ionic radius is the mechanism allowing to allocate selectively both types of metal ion within the molecular structure, exploited with the simultaneous use of two β-diketone-type ligands. To assist the photophysical investigation of this heterometallic species, the analogues [YbLaYb] (**2**) and [LuNdLu] (**3**) have also been prepared. Sensitization of Yb^3+^ and Nd^3+^ in the last two complexes, respectively, was observed, with remarkably long decay times, facilitating the determination of the Nd-to-Yb ET within the [YbNdYb] composite. This ET was demonstrated by comparing the emission of iso-absorbant solutions of **1**, **2**, and **3** and through lifetime determinations in solution and solid state. The comparatively high efficiency of this process corroborates the facilitating effect of having two acceptors for the nonradiative decay of Nd^3+^ created within the [YbNdYb] molecule.

## Abstract 2

A distributive Nd-to-Yb ET phenomenon, from one donor to two acceptors, has been described following the preparation and study of three pure hetero-lanthanide complexes exhibiting sequences [YbNdYb], [YbLaYb], and [LuNdLu] of closely lying metals.

## Introduction

**Figure 1:**
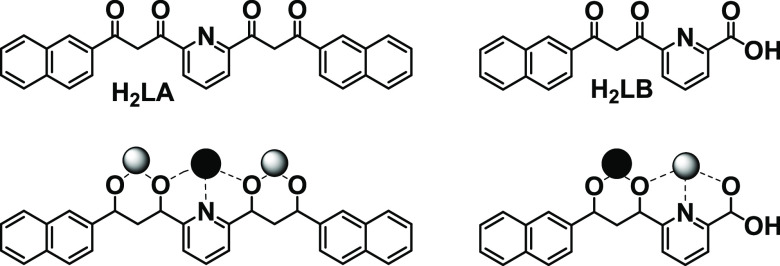
(Top) Molecular structure of ligands 2,6-bis[(3-oxo-3-naphthalene-2-yl)propionyl]pyridine (H_2_LA) and 6-(3-(naphthalene-2-yl)-3-oxopropanoyl)-picolinic acid (H_2_LB). (Bottom) Coordination modes of LA^2–^ and LB^2–^ within compounds **1**, **2**, and **3**. Black and gray balls are two different Ln^3+^ ions.

The electronic structure of lanthanides gives place to energy ladders featuring long-lived excited states that originate sharp 4f–4f emission bands spanning a large range of wavelengths (from the near-infrared (NIR) to the UV and visible region).^1,2^ This engenders photoluminescence properties that find applications in a wide variety of fields. The latter include medical imaging and bioassays,^3^ solar energy harvesting,^4−6^ data transmission and telecommunications,^7^ or light-emitting devices.^8^ These Laporte forbidden intraconfigurational 4f–4f transitions exhibit long radiative emission lifetimes as well as reduced absorption coefficients, restricting the access to the emitting excited states *via* direct light absorption. These may be, however, easily attained following the efficient energy transfer (ET) from nearby excited species called antennae, enabling then the light emission by the so-called sensitized luminescence process.^9,10^ Most often, ET takes place from excited π-conjugated organic moieties that usually transfer the energy to the Ln ion following a process of intersystem crossing (ISC).^3,11−19^ Sensitization may also take place by other Ln metal ions, previously lifted to (4f)* excited states.^20,21^ The Ln-to-Ln′ ET involved in this sensitization allows for very interesting up- or down-conversion processes.^22^ Up-conversion leads to emission of photons of higher energy than the photons absorbed (anti-Stokes process).^4,23−25^ It occurs when the emissive center is capable of accumulating energy from more than one ET event, and its advantages make it promising for new applications such as up-conversion lasers,^26,27^ improved solar cells,^5,28^ or bio-probes for theranostics.^29^ Down-conversion in turn, also called quantum cutting, produces photons of lower energy than the incoming ones.^5,28^ When more than one photon per absorption event is emitted, >100% quantum yields may be observed.^30,31^ Accessing improved solar cells may be among the benefits of this phenomenon.^32^ The active components leading to sensitized luminescence may be exploited in a variety of forms (glasses and ceramic materials,^33^ nanoparticles,^20^ polymers,^34^ metal–organic frameworks,^35^*etc.*). In this respect, an optimal mode of implementing it is building up the antenna and the emissive center into discrete molecules.^10,12,36−38^ This is advantageous because chemical synthesis and design allow incorporating a large variety of organic sensitizers, tuning the distances between energy donors and acceptors, incorporating other functional properties, or tailoring the molecular properties for their processability. In this regard, probing Ln-to-Ln′ (Ln ≠ Ln′) ET within molecules is not an easy task because it requires the preparation of pure heterometallic species with the different Ln metal ions positioned selectively at specific locations of the molecular architecture.^39,40^ Since the 4f valence electrons of lanthanides are strongly shielded by 5p and 5s electrons, they exhibit very similar reactivity; therefore, site-selective heterometallic Ln molecules are generally obtained *via* sequential methodologies that are very tedious.^41,42^ These include (i) step-by-step deprotection/opening of coordination sites,^43^ (ii) covalent linkage of different preformed coordination complexes,^44−50^ (iii) sequential activation and complexation of coordination sites,^51−58^ or (iv) enantiomeric self-recognition and mutual binding of chiral components.^59^ Some of the valuable molecules obtained in these manners have afforded the opportunity to study for the first time interesting intramolecular Ln-to-Ln′ ET phenomena,^41^ such as Tb-to-Yb,^60,61^ Tb-to-Er,^62^ or Dy-to-Tb.^47^ On the other hand, one-step reactions of self-assembly usually lead to mixtures of metal distributions within the molecule, sometimes close to statistical.^63−65^ This has been used in trinuclear complexes to combine three metals in variable proportions within one molecule and tune the optical properties of the bulk mixture by modulating the proportions of the three metals employed in the reaction.^66^ The method also allowed identification of intramolecular Nd-to-Yb ET within [YbNdYb] molecules, with the inconvenient presence of molecules with the same structure but with other metal composition (*e.g.* [YbYbYb]).^67^ The selectivity of the thermodynamically controlled synthetic procedures relies on the ability to discriminate the various metals capitalizing on their differences in ionic radius (*r*_Ln_). Some years ago, we discovered a ligand system capable of coordinating with remarkable selectivity two different metals within heterometallic [LnLn′] molecules through the generation of two coordination sites, one binding the larger ion and the other one the smaller ion. The reason for this segregation is that the larger site features two “O,N,O” (dipicolinate type) tridentate chelates and one “O,O” diketonate pocket, whereas the smaller position involves one tridentate and two bidentate pockets.^68−71^ More recently, we have reported that combining two ligands, both featuring dipicolinate (O,N,O) and diketonate (O,O) units (H_2_LA, 2,6-bis[(3-oxo-3-naphthalene-2-yl)propionyl]pyridine; H_2_LB, 6-(3-(naphthalene-2-yl)-3-oxopropanoyl)-picolinic acid; Figure 1), produces a novel molecular architecture when mixed with certain combinations of two different Ln(NO_3_)_3_ salts positioning selectively both metals in a [LnLn′Ln] topology.^72^

All of these trinuclear complexes exhibit the formula [Ln_2_Ln′(LA)_2_(LB)_2_(H_2_O)_2_(py)](NO_3_), (py = pyridine), where *r*_Ln′_ > *r*_Ln_. Here, the central position of the molecular scaffold is made of two “O,N,O” dipicolinate units and two “O,O” diketonates, favoring the coordination of larger metal ions. In turn, the external positions are conformed by two “O,O” and one “O,N,O” pockets, respectively, thus generating shorter bond distances to Ln. Mass spectrometry (MS) and density functional theory (DFT) calculations indicated that, for all of the compounds of this family reported so far, the selectivity and purity of the heterometallic molecules is unparalleled and extremely high.^73,74^ We report here the synthesis and structure of the new compound [Yb_2_Nd(LA)_2_(LB)_2_(H_2_O)_2_(py)](NO_3_) (**1**), also noted [YbNdYb], prepared to investigate the possible intramolecular Nd-to-Yb ET, exploiting a potential distributive mechanism to improve the ET probability and thus the efficiency of the process. Complex **1** provides this opportunity by featuring one Nd^3+^ and two Yb^3+^ centers in one molecule that can be produced in pure form. The unparalleled selectivity and the versatility of this synthetic resource enabled the preparation of the analogues [Yb_2_La(LA)_2_(LB)_2_(H_2_O)_2_(py)](NO_3_) (**2**) and [Lu_2_Nd(LA)_2_(LB)_2_(H_2_O)_2_(py)](NO_3_) (**3**). Complexes **2** and **3** were obtained to assist the interpretation of the photophysical properties of the [YbNdYb] system (La and Lu play as non-emitting centers) allowing the comparative study of the optical properties of each active metal (Yb and Nd) without the influence of the other.

## Results and Discussion

### Synthesis and Structures

The new molecules [Yb_2_Nd(LA)_2_(LB)_2_(H_2_O)_2_(py)](NO_3_) (**1**), [Yb_2_La(LA)_2_(LB)_2_(H_2_O)_2_(py)](NO_3_) (**2**), and [Lu_2_Nd(LA)_2_(LB)_2_(H_2_O)_2_(py)](NO_3_) (**3**) were prepared using the same general procedure (see Supporting Information (SI)). Equimolar quantities of H_2_LA and H_2_LB were mixed in pyridine with stoichiometric amounts of Ln(NO_3_)_3_ (Ln = Yb or Lu) and Ln′(NO_3_)_3_ (Ln′ = Nd or La), *i.e.*, in the Ln/Ln′ molar ratio 2/1, together with 1 equiv of CuCl_2_. The latter salt is not strictly necessary to propose a chemical reaction of formation of complex **1**, **2**, or **3** (see below), but its presence was in all cases found to be indispensable to obtain the crystals of the target complexes, most probably as modulators of the process of self-assembly and/or that of crystallization.^75^ A reaction including the “side” participation of this modulator is shown in the equation below (eq 1).
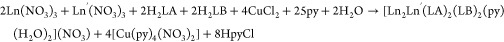
1

**Figure 2:**
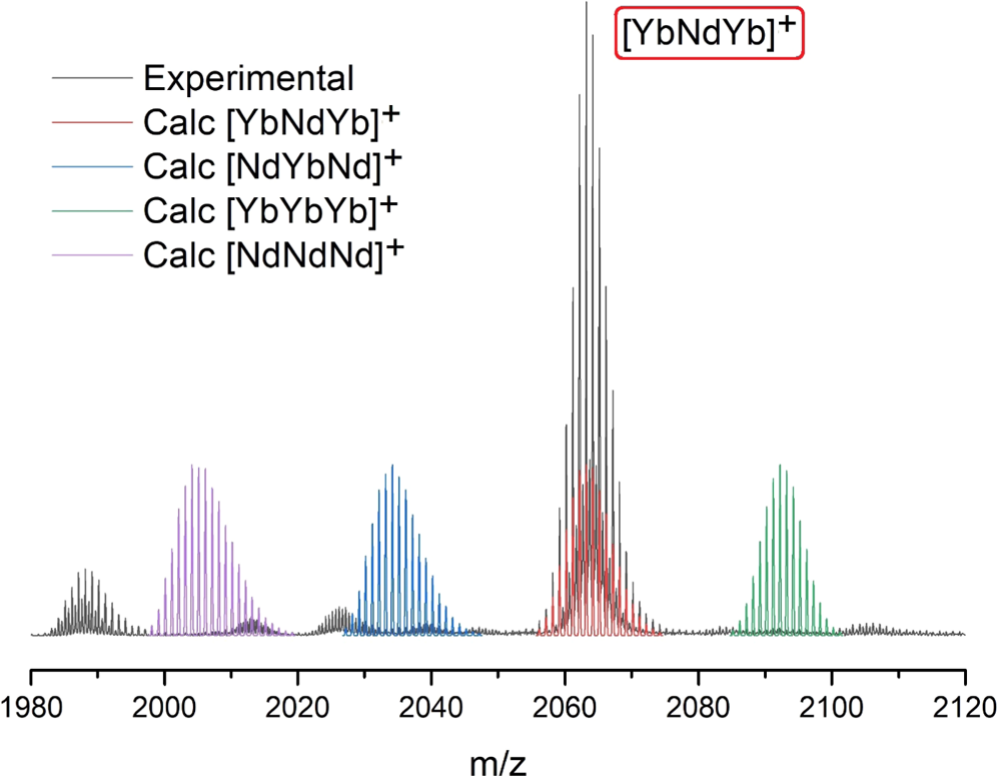
Selected region of the experimental (black line) ESI-MS spectrogram of compound **1** ([YbNdYb]), emphasizing the [YbNdYb(LA)_2_(LB)_2_]^+^ (*m*/*z* = 2063.1739) fragment, together with the corresponding calculated signals for the [NdNdNd] (purple line), [YbNdNd] (blue line), [YbNdYb] (red line), and [YbYbYb] (green line) metal distributions.

Large yellow crystals of the [LnLn′Ln] complexes (**1**, **2** or **3**) were obtained by diffusing a layer of heptane into the reaction mixture over the course of a month. These grew simultaneously with green crystals of [Cu(py)_4_(NO_3_)_2_], which were easily separated manually. The purity of the coordination compounds, once separated from the Cu^2+^ salt was established through various methods. The three compounds furnished satisfactory C,H,N microanalysis results, and most importantly, the metal composition from inductively coupled plasma (ICP) data fitted very well the proposed formulation (found Ln′/Ln molar ratios of 0.51, 0.52, and 0.51, for **1**, **2**, and **3**, respectively). Support for the bulk homogeneity of the solid-state polycrystalline samples is given by the IR spectra, almost identical for **1**, **2**, and **3** (Figure S1). In solution, positive electrospray ionization mass spectrometry (ESI-MS) strongly supported the purity of the [LnLn′Ln] moieties, present with strong signals in the absence of signals expected from any metal scrambling (Figures 2 and S2–S10).

**Figure 3:**
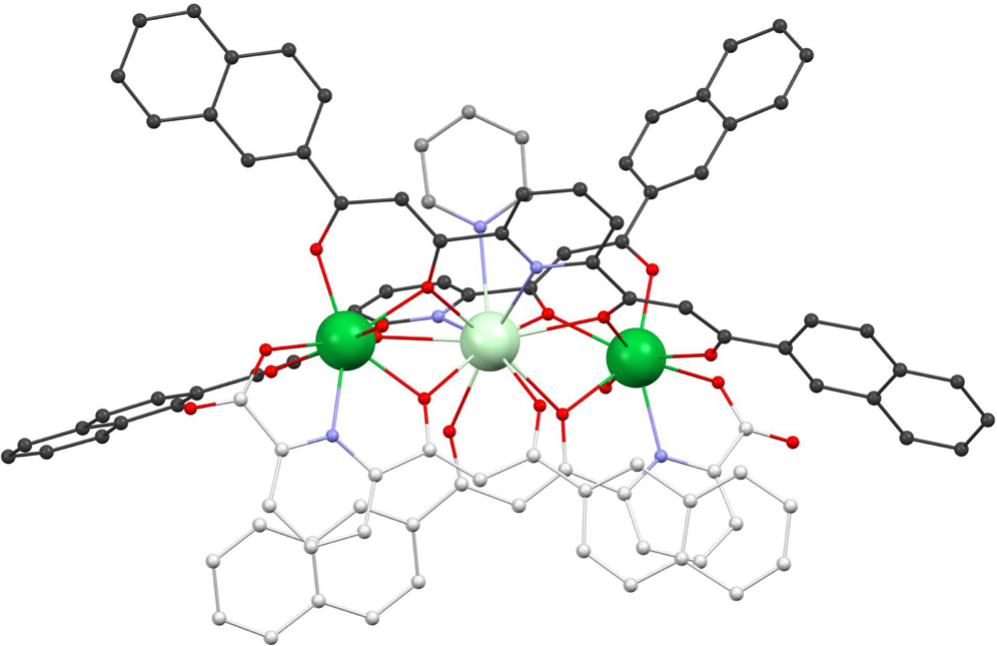
Molecular structure of the complex cation of [Yb_2_Nd(LA)_2_(LB)_2_(py)(H_2_O)_2_](NO_3_) (**1**). Colors: dark green, Yb; light green, Nd; red, O; dark gray, C from LA^2–^; gray, C from py; light gray, C from LB^2–^; purple, N. Hydrogen atoms not shown. Complexes from **2** and **3** are isostructural.

Compounds **1**, **2**, and **3** are virtually isostructural (only differing on the exact amount and disorder of the pyridine lattice solvent molecules). Therefore, the structures of the three compounds will be described here jointly. Complexes **1**, **2**, and **3** crystallize in the triclinic space group *P*1̅. In all cases, the asymmetric unit is composed of one [Ln_2_Ln′(LA)_2_(LB)_2_(py)(H_2_O)_2_]^+^ complex cation (LnLn′Ln = YbNdYb, **1**; YbLaYb, **2**; LuNdLu, **3**; Figures 3 and S11–S13), one NO_3_^–^ counterion, and 11/10/10 (in the **1**/**2**/**3** format) pyridine molecules of crystallization. The unit cell contains two such asymmetric units (Tables S1–S3).

The [LnLn′Ln] complex (Figure 3) is cemented by two LA^2–^ ligands bridging the three metals in the μ_3_ mode and two μ_2_-LA^2–^ donors (Figure 1) linking the central ion with one of the peripheral metals. All ligands chelate each of the metals to which they coordinate with either one O,O diketonate group or one O,N,O tridentate pocket. Thus, the central metal is bound to two O,N,O groups and two O,O pockets with a coordination number of 11 completed by a pyridine ligand while each of the side metals feature two O,O and one O,N,O chelate in addition to one bound molecule of H_2_O, leading to a coordination number of eight. This distribution of chelating pockets generates two distinct coordination sites within the molecular scaffold, the central one favoring the binding of larger metals than the other. This is observed in the structural parameters, with average Ln–O and Ln′–O distances (in the format Ln1/Ln2, Ln′ for [Ln1Ln′Ln2]) of 2.30(2)/2.30(2), 2.60(3), 2.31(4)/2.30(3), 2.63(5), and 2.31(4)/2.30(4), 2.60(5) Å for complexes **1**, **2**, and **3**, respectively. The different sizes of both types of coordination sites are attributed to different distributions of O,O and O,N,O coordination pockets in them. Thus, the position with a larger ratio of tridentate *vs* bidentate pockets favors longer Ln–O bonds, as previously observed on the related family of heterometallic [LnLn′] complexes.^69,70,76^ The ideal polyhedrons that best describe the coordination geometry around the metal centers were determined through SHAPE calculations.^77^ The central metals (coordination number (CN) = 11) are best represented by a capped pentagonal antiprism (*C*_5*v*_) with distances to it of 6.229, 6.217, and 6.275 for **1**, **2**, and **3**, respectively. The peripheral ions (CN = 8) are most similar to a biaugmented trigonal prism (C_2*v*_), with distances to this ideal form, in the Ln1/Ln2 format, of 1.474/1.387, 1.509/1.301, and 1.270/1.509, respectively. The intramolecular Ln···Ln′ and Ln···Ln distances (in the format Ln1···Ln′/Ln2···Ln′, Ln1···Ln2 for [Ln1Ln′Ln2]) are 3.9294(5)/3.9313(5), 7.851(1), 3.9424(6)/3.945(6), 7.880(1), and 3.934(2)/3.932(2), 7.857(2) Å for complexes **1**, **2**, and **3**, respectively. The three metals within the complex are almost linearly arranged, with angles slightly below 180° (174.34, 174.79, and 174.29°). Within the lattice, the complex cations interact pairwise through two complementary [O···H–O] hydrogen bonds with two H_2_O ligands from either complex acting as donors and the carboxylate coordinating O-atoms of two LB^2–^ ligands being the acceptors. This causes the external Ln ions to be closer among two different molecules (intermolecular Ln···Ln shortest separations of 6.086, 6.057, and 6.063 Å) than within the [LnLn′Ln] complexes (see above).

### Photophysical Properties

The availability of complexes **1**, **2**, and **3** as pure phases with metal distributions [YbNdYb], [YbLaYb], and [LuNdLu] was exploited to investigate the potential intramolecular ET between Yb^3+^ and Nd^3+^ in **1**, where Nd^3+^ is surrounded by two Yb^3+^ ions within the molecule. We had previously demonstrated similar Yb-to-Nd ET within a related dinuclear [NdYb] complex with a rather modest efficiency in MeOH of around 10%.^71^ To probe the Nd-to-Yb ET in the trinuclear analogue, an extensive photophysical investigation was carried out on the three complexes, both, in 10^–4^ M solution (1:1 mixture of MeOH and dimethyl sulfoxide (DMSO)) and in the solid state at room temperature (RT) and at 77 K. These studies included absorption, excitation, and emission spectra, as well as lifetime decay determinations. The main photophysical data are compiled in Table 1.

**Table 1:** Summary of Selected Photophysical Data of Ligands H_2_LA and H_2_LB, and of Complexes **1**, **2**, and **3**, Obtained in Diluted Solution (1:1 Mixture of MeOH and DMSO) at Room Temperature^d^

	λ_abs(max)_ (nm)	ε (L mol^–1^ cm^–1^)	λ_em_^a^ (nm)	T_E_^b^ (cm^–1^)	τ_obs_ (μs)	Φ_ET_^c^
H_2_LA	350	12 700	455	18 950		
H_2_LB	368	11 250	435	19 050		
YbNdYb (**1**)			976		9.3	
			1065		0.24	0.87
YbLaYb (**2**)			976		9.1	
LuNdLu (**3**)			1065		1.8	

a λ_exc_ = 400 nm.

b Energy of the triplet excited state measured at the 0-phonon transition marked as X in Figure 4.

c Excited-state lifetime.

d Efficiency of the Nd–Yb energy transfer.  (eq 2).

The energy transfer process from the excited states of Nd^3+^ to Yb^3+^ was studied following strategies previously reported by us on bimetallic coordination compounds.^71^ The emission properties of the derivatives [YbLaYb] (**2**) and [LuNdLu] (**3**) were studied first to confirm the antenna effect of the ligands on the Yb^3+^ and Nd^3+^ centers, respectively, and study their luminescence characteristics. Subsequently, the composite system [YbNdYb] (**1**) was investigated to probe the potential ET from the ^4^F_3/2_ state of Nd^3+^ to the ^2^F_5/2_ state of the two neighboring Yb^3+^ centers. The first step was to calculate the energy of the triplet excited state (T_E_) of both organic antennae (H_2_LA and H_2_LB) by measuring their phosphorescence spectra (Figure 4). Deconvolution of the phosphorescent bands (at 77 K with a time delay of 0.05 ms) yielded the 0-phonon transition as the excitation to the lowest lying triplet state of the ligands at ∼18 950 and ∼19 050 cm^–1^ for H_2_LA and H_2_LB, respectively. The energy of both excited states is sufficiently high to sensitize the emission of the ^2^F_5/2_ state of Yb^3+^ (∼10 250 cm^–1^) and the ^4^F_3/2_ state of Nd^3+^ (∼11 260 cm^–1^).^78−80^ Therefore, emission spectra of Nd^3+^ and Yb^3+^ with main bands centered at 880/1060/1340 and 1000 nm, respectively, can be expected for compounds **2** and **3**.

**Figure 4:**
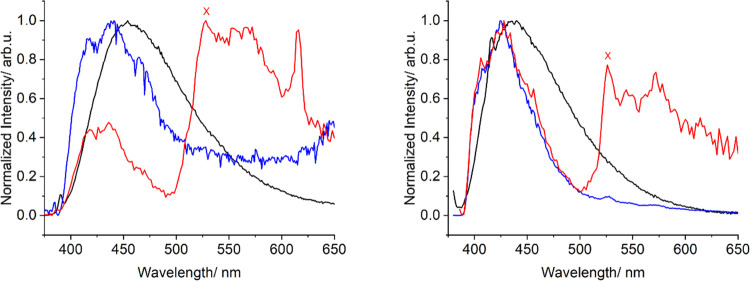
Normalized emission spectra (λ_exc_ = 400 nm) of H_2_LA (left) and H_2_LB (right) at room temperature (black trace), 77 K (blue trace), and 77 K with a 0.05 ms delay (red trace). X indicates the position of the lowest triplet state.

**Figure 5:**
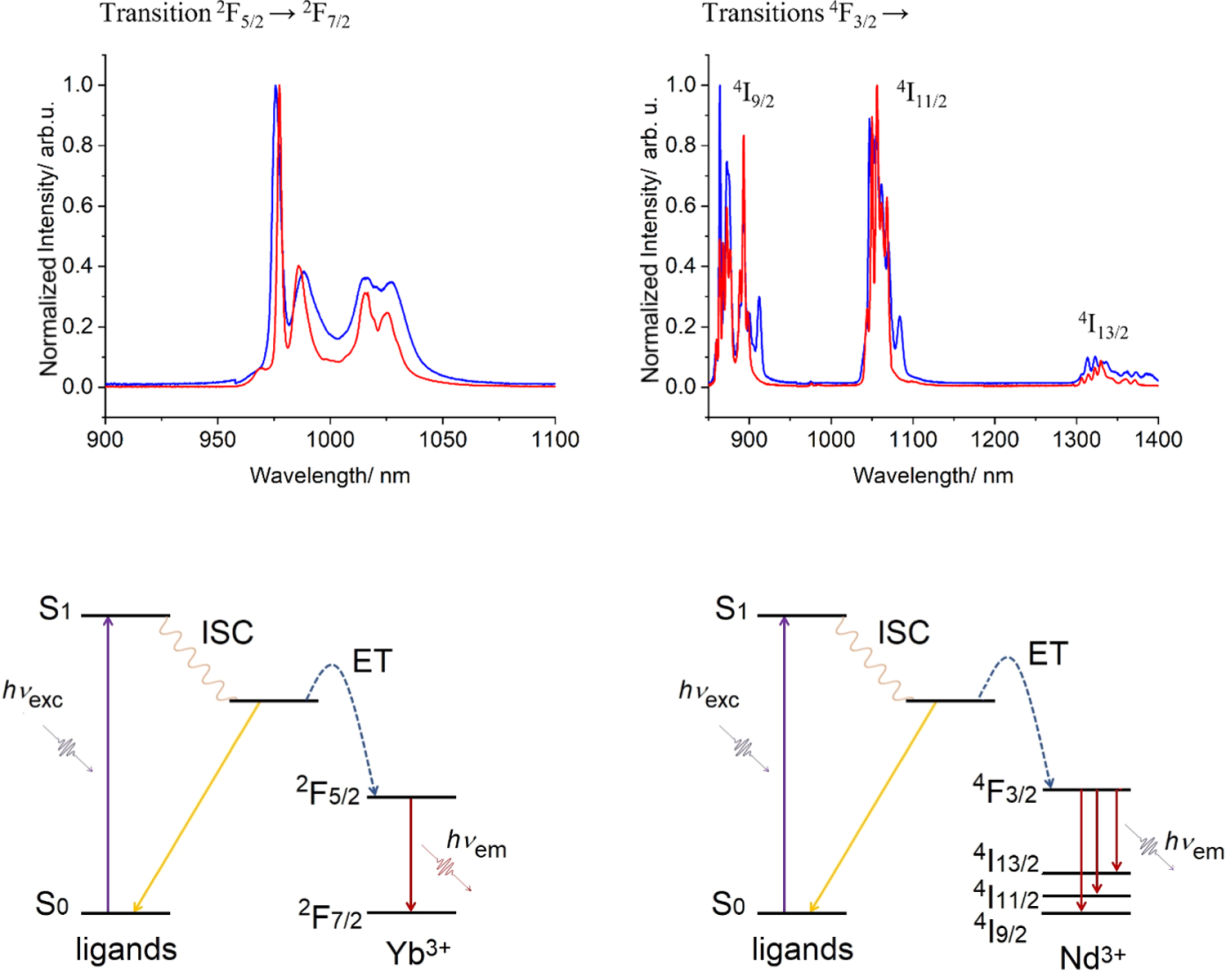
Normalized emission spectra (λ_exc_ = 400 nm) of (left) [YbLaYb] (**2**) and (right) [LuNdLu] (**3**) comparing the respective responses in diluted solution MeOH/DMSO (1:1) at 77 K (red traces) with these in the solid state (blue traces). At the bottom are the diagrams representing the emission processes. Detailed deconvolutions into transitions to the various Kramer’s doublets are given at the SI (Figures S15 and S17).

This potential sensitization was investigated first on [YbLaYb] (**2**). The photoluminescence spectrum of this compound indeed exhibited the expected Yb^3+^ emission with a maximum at 980 nm, which could be assigned to the ^2^F_5/2_ → ^2^F_7/2_ transition of this metal, both in solution and the solid state (Figure S14). When these spectra were measured at 77 K, the emission bands were considerably narrower due to the reduced inhomogeneous line broadening and to the decreased contribution of “hot bands” (*i.e.* emission bands from thermally populated *m*_*J*_ excited states). This enhanced resolution revealed in both cases a single set of four well-defined bands corresponding to the four expected sublevels of the Kramers ^2^F_7/2_ ground state, indicating that both Yb^3+^ emissive centers are identical (Figures 5 and S15). The spectral features of the ground state in the solid state and in solution are identical, suggesting that the structure of the complex determined in the solid state by single crystal X-ray diffraction (SCXRD) is preserved in solution (Figure 5). As previously demonstrated for Yb^3+^ complexes, the total crystal field splitting (Δ_CFS_) within the ^2^F_7/2_ ground state is a key signature of its coordination symmetry.^81,82^ In **2**, Δ_CFS_ = 580 and 530 cm^–1^ in frozen solution and in the solid state, respectively, were found to be slightly higher than in previously reported examples with *D*_4*h*_ symmetry (∼500 cm^–1^),^83−85^ suggesting a scarcely lower symmetrical environment. This is consistent with the triangular dodecahedron with *C*_2*v*_ symmetry around both Yb^3+^ centers revealed by the SCXRD experiments. Additionally, the excited-state lifetime decay measured at 976 nm in MeOH/DMSO (1:1) solution at room temperature was satisfactorily fitted with a monoexponential function. The found value of 9.1 μs is considerably longer than the 2 μs obtained with the previously published [LnYb] complexes (Ln = Eu, Nd, Gd) in MeOH at room temperature.^71^ This enhancement suggests a more rigid environment in [YbLaYb] (**2**), which prevents nonradiative deactivation processes.

On the other hand, the luminescence spectra for complex [LuNdLu] (**3**) yielded the characteristic Nd^3+^ bands coming from the ^4^F_3/2_ → ^4^I_*J*_ (*J* = 9/2, 11/2, 13/2) transitions, with maxima emission wavelengths at 910, 1060, and 1350 nm, respectively (Figures 5 and S16), both in solution and the solid state. Comparing the emission spectra at room temperature and 77 K, the fine splitting of the emission bands due to the ligand field effect is revealed. Furthermore, taking into consideration the Kramers doublets, a maximum number of peaks of 5, 6, and 7, respectively, could be observed for the three transitions. In fact, a careful examination of the spectra (Figure S17) reveals more peaks than expected for each transition, each time in form of smaller contributions at the higher-energy side. This suggests that hot band emissions from the *m*_*J*_ excited states of the ^4^F_3/2_ term are still present even at 77 K.^86^ While the hot state’s contribution can explain the presence of additional peaks, vibronic transitions cannot be excluded. Otherwise, the similarity between emission spectra of the solid state and solution at low temperature suggests the persistence of the geometry of the first coordination sphere of the complex upon dissolution. The excited-state lifetime value at 1056 nm in solution at room temperature was found to be 1.7 μs. Consistent with complex **2**, in [LuNdLu] (**3**) the decay is much longer than that previously observed for the [NdLu] dimer analogue, found to be 178 ns in MeOH.^71^

**Figure 6:**
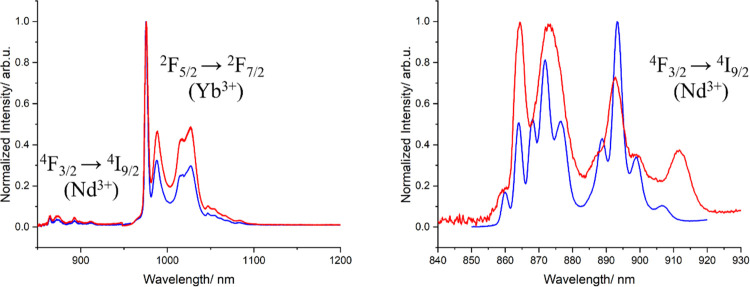
Normalized emission spectra (λ_exc_ = 400 nm) of [YbNdYb] (**1**), comparing the response in diluted frozen solution MeOH/DMSO (1:1) (red trace) and in the solid state at 77 K (blue trace) between (left) 850 and 1200 nm and (right) 840 and 930 nm, respectively, to highlight the contribution of the ^4^F_3/2_ → ^4^I_9/2_ emission of Nd^3+^. Curves from both regions have been obtained from different measurements; therefore, the normalized intensities are not mutually comparable.

The composite complex [YbNdYb] (**1**) shows emission from both Yb^3+^ and Nd^3+^ in all conditions studied (Figures 6 and S18). A superficial inspection of the spectrum reveals that it is strongly dominated by the Yb^3+^ emission, whereas the Nd^3+^ contribution can only be detected upon zooming. At low temperatures, the fine CFS of both emitters can be clearly measured.

As observed for [YbLaYb] (**2**), when moving from room temperature to 77 K, the hot bands of the Yb^3+^ ions in [YbNdYb] (**1**) vanish and the fine splitting becomes much better defined (Figure S19), allowing the calculation of the Δ_CFS_, found to be 500 and 528 cm^–1^ in frozen solution and solid state, respectively. These values are consistent with those obtained for [YbLaYb] (**2**) (Figure S20) and with the experimental solid-state structure, which also features a triangular dodecahedron coordination geometry of the Yb^3+^ centers. Regarding the Nd^3+^ emission lines, the ^4^F_3/2_ → ^4^I*_J_* (*J* = 9/2 and 13/2) transitions are easily observable while the ^4^F_3/2_ → ^4^I_11/2_ emission overlaps with the bands for the ^2^F_5/2_ → ^2^F_7/2_ process of Yb^3+^. The Nd^3+^ emission of **1** is identical to that observed for complex **3** (Figure S21).

**Figure 7:**
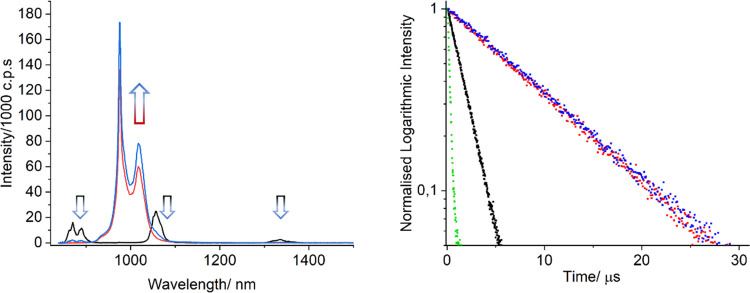
Emission spectra (λ_exc_ = 400 nm) of iso-absorbant solutions of complexes [YbLaYb] (**2**) (red trace), [LuNdLu] (**3**) (black trace), and [YbNdYb] (**1**) (blue trace) in MeOH/DMSO (1:1) solution at room temperature (left) and their excited states decay (right) measured at 976 nm for **2** (red trace) and **1** (blue trace) and at 1056 nm for **3** (black trace) and **1** (green trace).

The studies on complexes [YbLaYb] (**2**) and [LuNdLu] (**3**) described above demonstrate that ligands LA^2–^ and LB^2–^ efficiently sensitize the Yb^3+^ and Nd^3+^ emission. This stimulated an investigation of the possible ET from the Nd^3+^(^4^F_3/2_) to the Yb^3+^(^2^F_5/2_) excited state within [YbNdYb] (**1**). With this aim, iso-absorbant solutions at 400 nm (*i.e.*, with identical absorbance, *A* = 0.4, at this wavelength) of complexes **1**, **2**, and **3** were prepared to compare their emission spectra (Figure 7). Revealingly, the Nd^3+^ emission of [YbNdYb] (**1**) strongly decreases with respect to [LuNdLu] (**3**) while the Yb^3+^ emission slightly increases in comparison to [YbLaYb] (**2**), strongly suggesting the Nd-to-Yb ET. Lifetime determinations were carried out to corroborate this hypothesis. The lifetimes of [YbNdYb] (**1**) measured in solution (RT) at 976 and 1056 nm wavelengths (for Yb^3+^ and Nd^3+^, respectively) were fitted to monoexponential decay models, yielding values of 9.3 and 0.24 μs for the Yb^3+^ and Nd^3+^ emission, respectively (Figure 7). These data clearly show a dramatic reduction of the Nd^3+^ lifetime of **1** compared to [LuNdLu] (**3**) and a conservation of the Yb^3+^ one compared to [YbLaYb] (**2**). This behavior is characteristic of an efficient Nd-to-Yb energy transfer that acts as an additional non radiative deexcitation pathway for the Nd^3+^ emitter. Using eq 2 (see footnote “d” of Table 1), the efficiency of the transfer could be quantified to 87%. This rate of ET efficiency is much improved with respect to the previously reported dimer (10% in MeOH).^71^ This improvement could be related to the presence of two acceptors (Yb^3+^) for a single donor (Nd^3+^) in the present trinuclear system, in a similar manner to that previously reported for a family of supramolecular [CrLnCr] compounds,^87^ where the rare earth was acting as acceptor of both Cr ions. However, one cannot dismiss the possible effect of having a more rigid structure, which should lead to a reduction of the nonradiative deactivation processes. To further corroborate the contribution of the distributive ET path towards the two Yb^3+^ acceptors, the lifetime measurements were studied in the solid state (conditions where the rigidity of the ligand scaffold plays a less important role). The lifetime decays of [LuNdLu] (**3**) and [YbNdYb] (**1**) measured at 1056 nm at room temperature were fitted to monoexponential decays models, giving values of 1.43 and 0.15 μs for the two complexes, respectively. These values translate to an efficiency rate of ET of 89%, being again much higher than the homologous published dimer in the solid state (55%), and therefore highlighting the importance of the higher probability of energy transfer in the trinuclear compounds with respect to the binuclear counterparts.^71^

**Figure 8:**
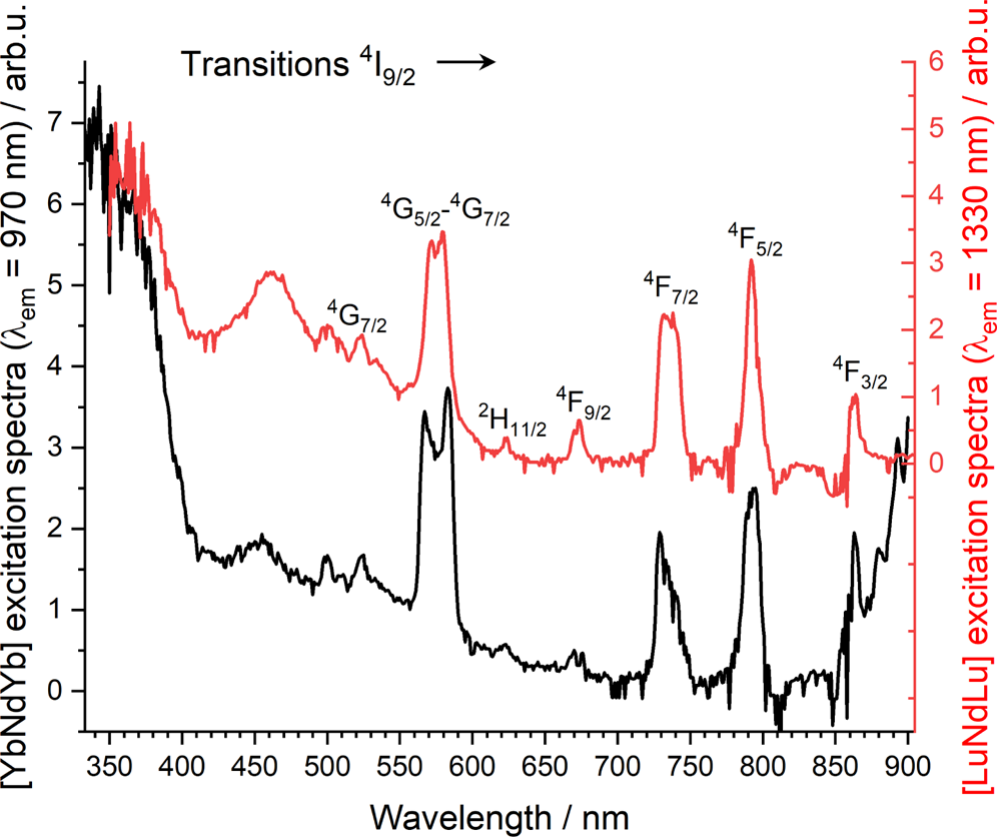
Excitation spectra of complex [LuNdLu] **3** (red trace) (λ_em_ = 1330 nm) and complex [YbNdYb] **1** (λ_em_ = 970 nm) in the solid state at 77 K.

The ET was characterized in further detail by means of transfer excitation spectra focused directly on f–f transitions. Data were first obtained on [LuNdLu] (**3**) in the solid state at 77 K focusing of the emission wavelength at 1330 nm. Doing so, the main excitation lines observed could be assigned to the transitions coming from the ^4^I_9/2_ to higher excited states (Figure 8) of Nd^3+^. On the contrary, for complex **1**, the emission wavelength was fixed at 970 nm (Yb^3+^ emission). The excitation spectrum shows identical lines to complex **3** (Nd^3+^ excitation), suggesting that there is, indeed, emission at 970 nm coming from the Nd^3+^ center, and therefore, the direct energy transfer Nd-to-Yb.

**Figure 9:**
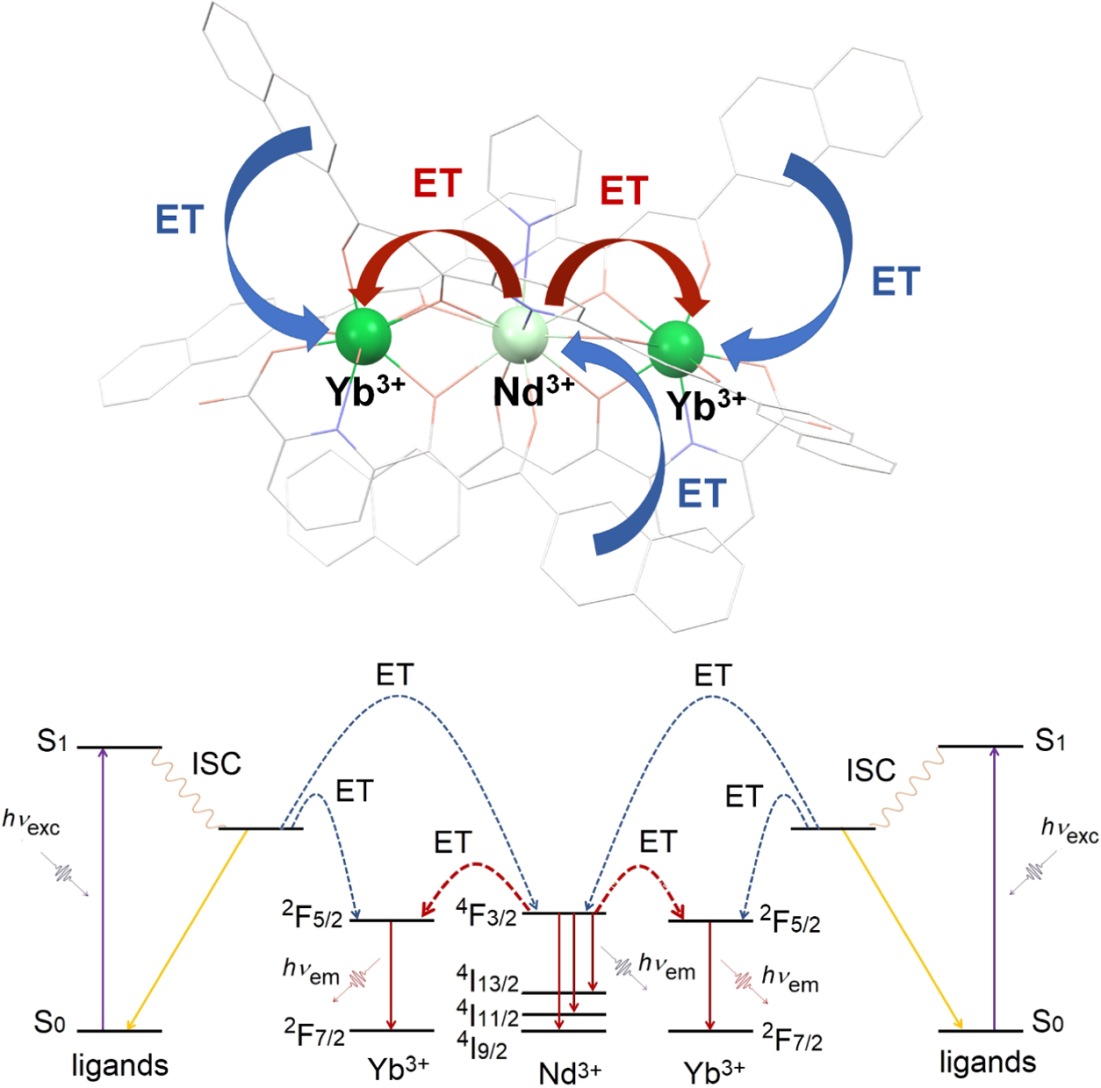
Schematic representation of the ET phenomena in complex [YbNdYb] **1** (top) and the corresponding terms and levels involved in these (bottom).

The photophysical events described above thus confirm the efficient ET from Nd^3+^ (one donor) to Yb^3+^ (two acceptors) within the same molecule, following ligand-based sensitization, as illustrated and summarized in Figure 9.

## Conclusions

The potential ET phenomena between Nd^3+^ and Yb^3+^ embedded in close proximity within a fixed and well-described molecular scaffold can be studied on a unique new family of [LnLn′Ln] heterometallic coordination constructs with formula [Ln_2_Ln′(LA)_2_(LB)_2_(H_2_O)_2_(py)](NO_3_), where LA^2–^ and LB^2–^ are two β-diketonate ligands. Specifically, the assemblies [YbNdYb] (**1**), [YbLaYb] (**2**), and [LuNdLu] (**3**) have been obtained and characterized by SCXRD, specifically to demonstrate intramolecular Nd-to-Yb ET. The metal distribution within **1** dramatically enhances the efficiency of this process due to the existence of two acceptors per donor. This efficient transfer occurs in solution as well as in the solid state. The resulting doubled pathway can be used as a strategy to increase the yield of the indirect sensitization of Yb^3+^ through excited Nd^3+^, contributing to the generation of efficient molecular devices for specific light wavelength conversions.

## Supporting Information Available

Synthetic procedures, experimental details of physical measurements, IR spectra and MS diagrams of all complexes, details, tables and figures of SCXRD molecular structures, and detailed emission spectra and analysis (PDF)
