# Supplementary material for: Distributive Nd-to-Yb Energy Transfer within Pure [YbNdYb] Heterometallic Molecules
Source: Inorg Chem. 2023 Feb 8;62(7):3106–15. doi: 10.1021/acs.inorgchem.2c03940 (PMC9945097; doi:10.1021/acs.inorgchem.2c03940)
Supplement: Supplementary file 1 — ic2c03940_si_001.pdf [file ic2c03940_si_001.pdf]

SUPPORTING INFORMATION

Distributive Nd-to-Yb Energy Transfer within Pure [YbNdYb] Heterometallic Molecules

Diamantoula Maniaki,[#,$] *Annika Sickinger,*[&] *Leoní Alejandra Barrios Moreno,*[#,$] *David Aguilà,*[#,$] *Olivier Roubeau,*[§] *Nicholas S. Settineri,*[%,+] *Yannick Guyot,*[x] *François Riobé,*[&] *Olivier Maury* [&] *Laura Abad Galán,*[‡,*] *and Guillem Aromí* [#,$,*]

[#] Departament de Química Inorgànica i Orgànica, Universitat de Barcelona, Diagonal 645, 08028 Barcelona, Spain. E-mail: aromi@ub.edu

[$] Institute of Nanoscience and Nanotechnology of the Univesity of Barcelona (IN2UB), Barcelona, Spain.

[‡] Departamento de Química Inorgánica, Universidad Complutense de Madrid, Avda. Complutense s/n 28040 Madrid, Spain. E-mail: E-mail: laabad03@ucm.es

[§] Instituto de Nanociencia y Materiales de Aragón (INMA), CSIC and Universidad de Zaragoza, Plaza San Francisco s/n, 50009, Zaragoza, Spain.

[%] Advanced Light Source, Berkeley Laboratory, 1 Cyclotron Road, Berkeley, California 94720, USA.

+ Department of Chemistry, University of California, Berkeley, Berkeley, CA 94720, USA

[&] Univ Lyon, ENS Lyon, CNRS, UMR 5182, Laboratoire de Chimie, F69342 Lyon, France.

[x] Univ. Lyon, Institut Lumière Matière, UMR 5306 CNRS–Université Claude Bernard, Lyon 1, 10 rue Ada Byron, F-69622 Villeurbanne Cedex, France.

Synthesis

Ligand H_2LA was prepared according to our published procedure.[1] Ligand H_2LB was prepared as previously reported by our group.[2]

[NdYb$_2$(LA)$_2$(LB)$_2$(py)(H$_2$O)$_2$](NO$_3$) (1). A yellow solution of H_2LA (10.2 mg, 0.032 mmol) and H_2LB (14.8 mg, 0.032 mmol) in pyridine (10 mL) was added dropwise under stirring to a light blue solution of $Nd(NO_3)_3·6H_2O$ (7 mg, 0.016 mmol), $Yb(NO_3)_3·5H_2O$ (14.4 mg, 0.032 mmol) and $CuCl_2·2H_2O$ (2.7 mg, 0.016 mmol) in pyridine (10 mL). The resulting green solution was left under stirring for 1 h and layered with heptane. After a week, green crystals of $[Cu(py)_4(NO_3)_2]$ had formed. After one month, large orange crystals of **1** were easily separated from the rest. Yield: ~15%. Elemental analysis (C,H,N), calcd. (found) for **1**·4H$_2$O: C, 54.52 (54.95); H 3.36, (2.99); N. 3.63 (4.15). Metal analysis (mols Nd/mols Yb, ICP), calcd. (found): 0.50 (0.51). MS: *m/z* = 2063.1739 $[NdYb_2(LA)_2(LB)_2]^+$, 1032.0892 $([NdYb_2(LA)_2(LB)_2]+H^+)^{2+}$, 1043.5749 $([NdYb_2(LA)_2(LB)_2]+Na^+)^{2+}$.

[LaYb$_2$(LA)$_2$(LB)$_2$(py)(H$_2$O)$_2$](NO$_3$) (2). Compound **2** was obtained as orange crystals (yield ~15 %) following the same synthetic procedure carried out for **1**, but using $La(NO_3)_3·6H_2O$ (7 mg, 0.016 mmol) instead of $Nd(NO_3)_3·5H_2O$. Yield: ~15%. Elemental analysis (C,H,N), calcd (found) for **2**·4.6H$_2$O: C, 54.39 (53.92); H 3.4, (2.92); N. 3.62 (3.74). Metal analysis (mols La/mols Yb, ICP), calc (found): 0.50 (0.52). MS: m/z = 2059.19 $[LaYb_2(LA)_2(LB)_2]^+$, 1030.09 $([LaYb_2(LA)_2(LB)_2]+H^+)^{2+}$, 1041.08 $([LaYb_2(LA)_2(LB)_2]+Na^+)^{2+}$.

[NdLu$_2$(LA)$_2$(LB)$_2$(py)(H$_2$O)$_2$](NO$_3$) (3). Compound **3** was obtained as orange crystals (yield ~15 %) following the same synthetic approach carried out for **1** but using $Lu(NO_3)_3·5H_2O$ (14.4 mg, 0.032 mmol) instead of $Yb(NO_3)_3·5H_2O$. Elemental analysis (C,H,N), calcd (found) for **3**·4.25H$_2$O: C, 54.33 (54.11); H 3.37, (3.06); N. 3.62 (3.9). Metal analysis (mols Nd/mols Lu, ICP), calcd. (found): 0.50 (0.51). MS: m/z = 2067.1647 $[NdLu_2(LA)_2(LB)_2]^+$, 1033.5924 $([NdLu_2(LA)_2(LB)_2]+H^+)^{2+}$, 1045.0863 $([NdLu_2(LA)_2(LB)_2]+Na^+)^{2+}$.

Other Physical Measurements

Elemental analyses were performed with a Perkin-Elmer Series II CHNS/O Analyzer 2400 (C, H, N) at the Servei de Microanàlisi of CSIC, Barcelona. IR spectra were recorded as KBr pellet samples on a Nicolet 5700 FTIR spectrometer.

Mass Spectrometry

Positive-ion ESI mass spectrometry experiments were performed by using a LC/MSD-TOF (Agilent Technologies) with a dual source equipped with a lock spray for internal reference introduction, at the Unitat d'Espectrometria de Masses from the Universitat de Barcelona. Experimental parameters: capillary voltage 4 kV, gas temperature

325°C, nebulizing gas pressure 103.42 kPa, drying gas flow 7.0 L min-1 and fragmentor voltage 175- 250 V. Internal reference masses were m/z 121.05087 (purine) or 922.00979 (HP-0921). Crystals of **1**, **2** or **3** were dissolved in mixtures of MeOH with the minimal amount of DMSO and introduced into the source by using a HPLC system (Agilent 110) with a mixture of H_2O/CH_3CN (1:1) as the eluent (200 µL min^{-1}). As observed previously for related clusters, the ionization caused the removal of both pyridine and water ligands from the complexes. For each complex, moieties related exclusively to the expected [LnLn'Ln] metal distribution were observed. Moreover, no signals for other metallic compositions were detected, thus evidencing not only the realization of the trinuclear heterometallic compound but also its robustness and exclusiveness in solution.

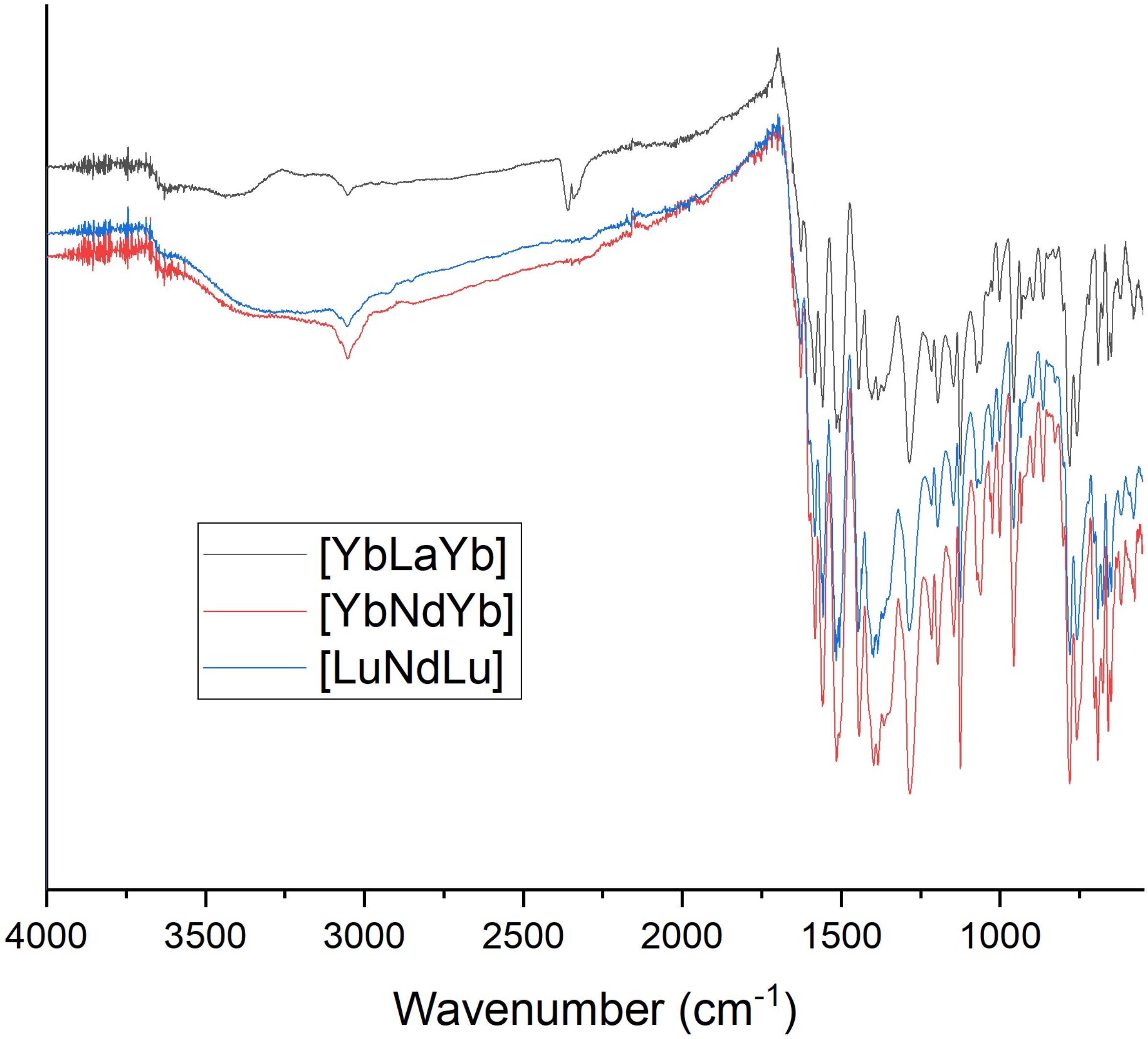

Figure S1. KBr pellet infrared spectrum of complexes **1** [YbNdYb], **2** [YbLaYb] and **3** [LuNdLu].

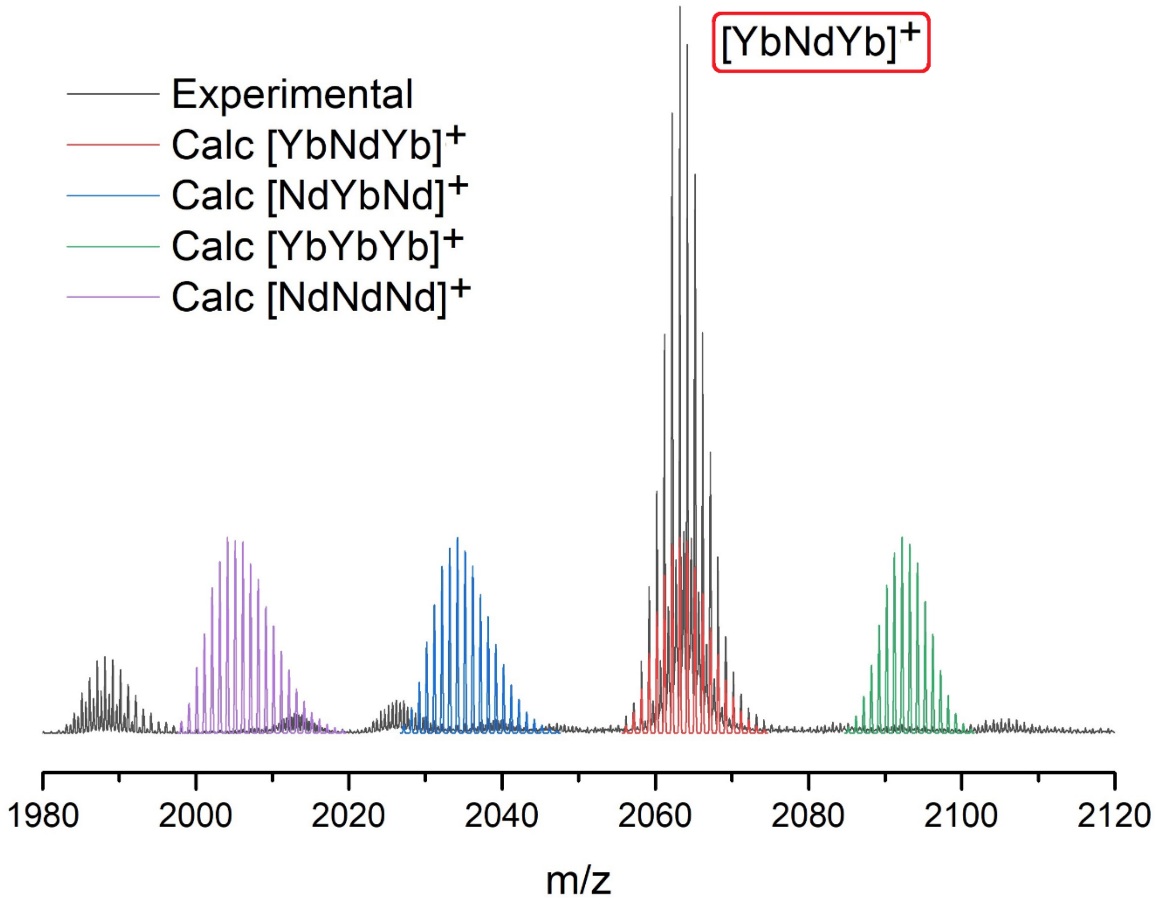

Figure S2. Selected region of the experimental (black line) ESI-MS spectrogram of compound **1** ([YbNdYb]), emphasizing the $[Yb_2Nd(LA)_2(LB)_2]^+$ (*m/z* = 2063.1739) fragment, together with the corresponding calculated signals for the $[Nd_3]$ (purple line), $[YbNd_2]$ (blue line), $[Yb_2Nd]$ (red line) and $[Yb_3]$ (green line), metal distributions.

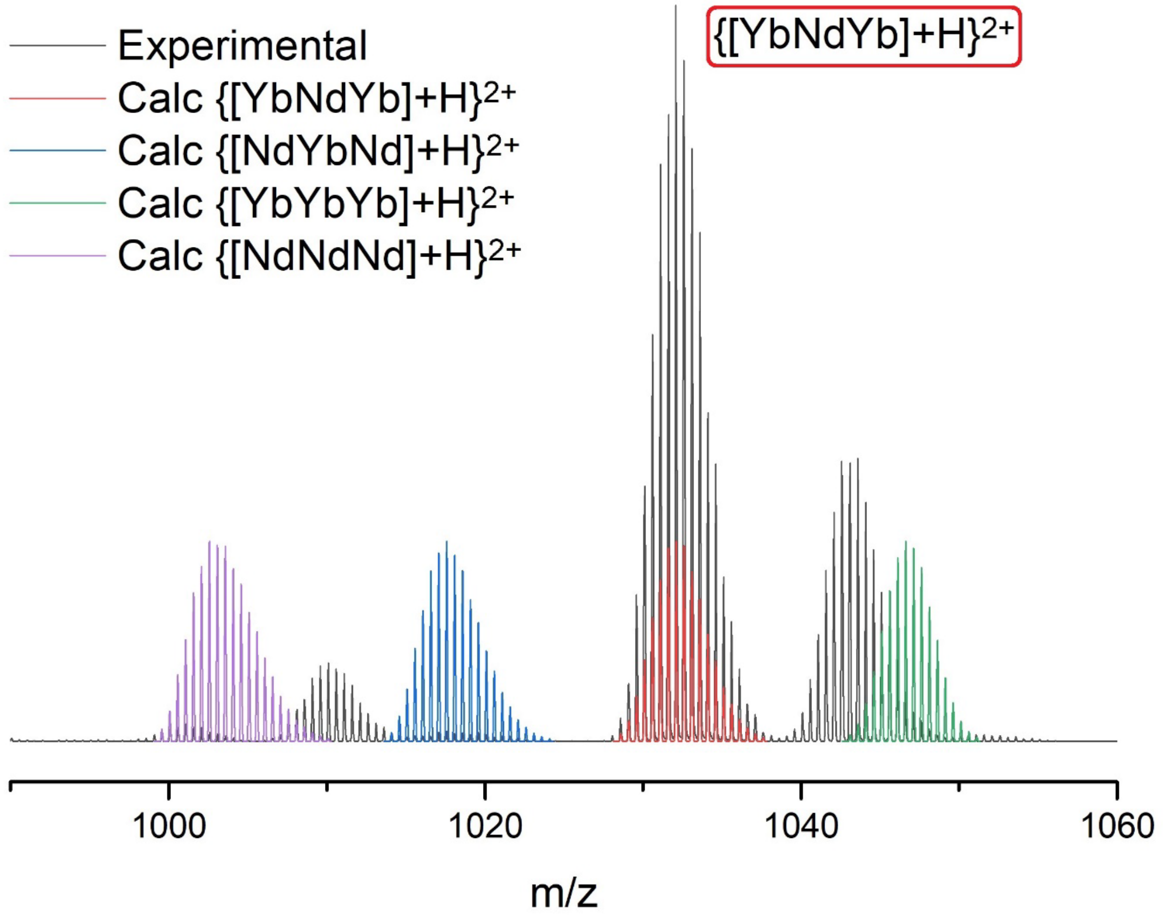

Figure S3. Selected region of the experimental (black line) ESI-MS spectrogram of compound **1** ([YbNdYb]), emphasizing the ($[Yb_2Nd(LA)_2(LB)_2]+H)^{2+}$ (*m/z* = 1032.0892) fragment, together with the corresponding calculated signals for the [Nd_3] (purple line), [$YbNd_2$] (blue line), [Yb_2Nd] (red line) and [Yb_3] (green line), metal distributions.

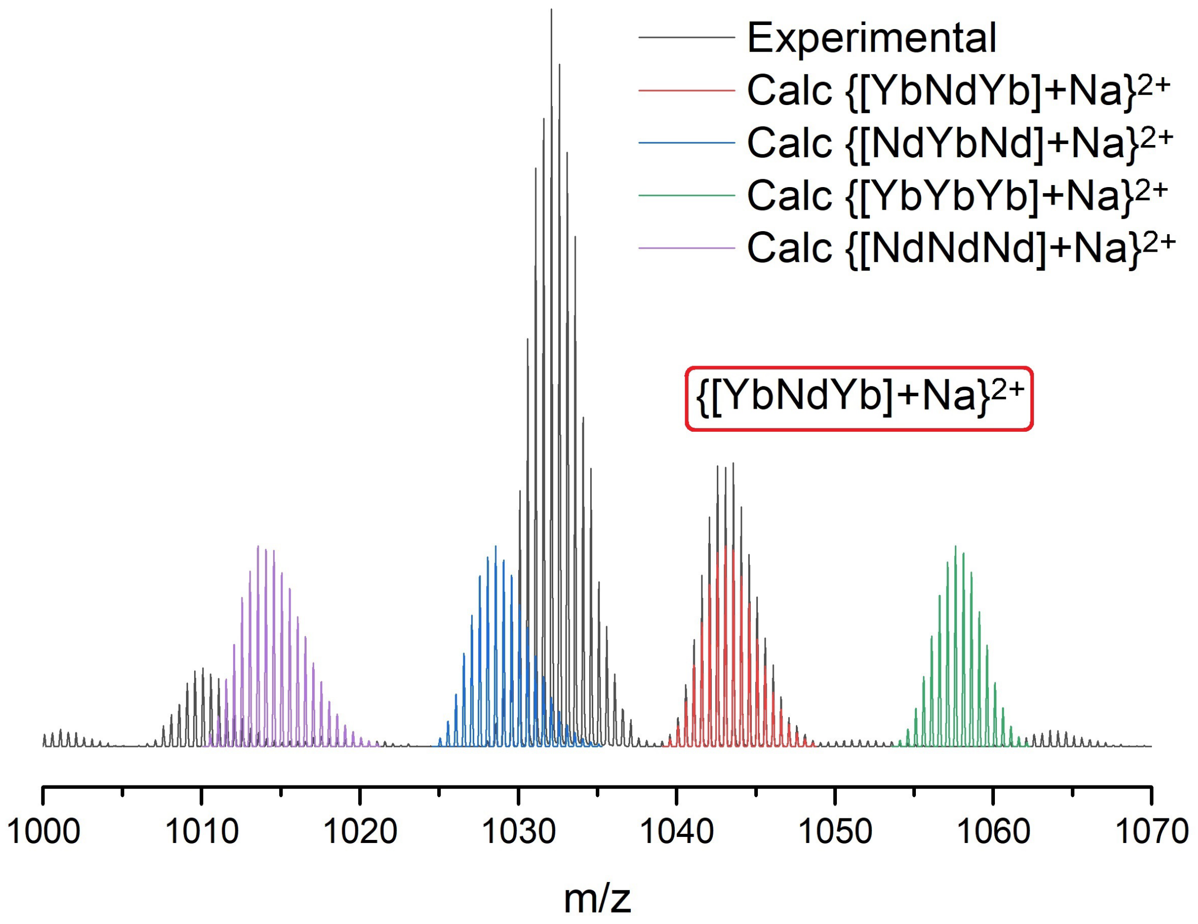

Figure S4. Selected region of the experimental (black line) ESI-MS spectrogram of compound **1** ([YbNdYb]), emphasizing the ([$Yb_2Nd(LA)_2(LB)_2$]+Na)$^{2+}$ (*m/z* = 1043.5749) fragment, together with the corresponding calculated signals for the [Nd_3] (purple line), [$YbNd_2$] (blue line), [Yb_2Nd] (red line) and [Yb_3] (green line), metal distributions.

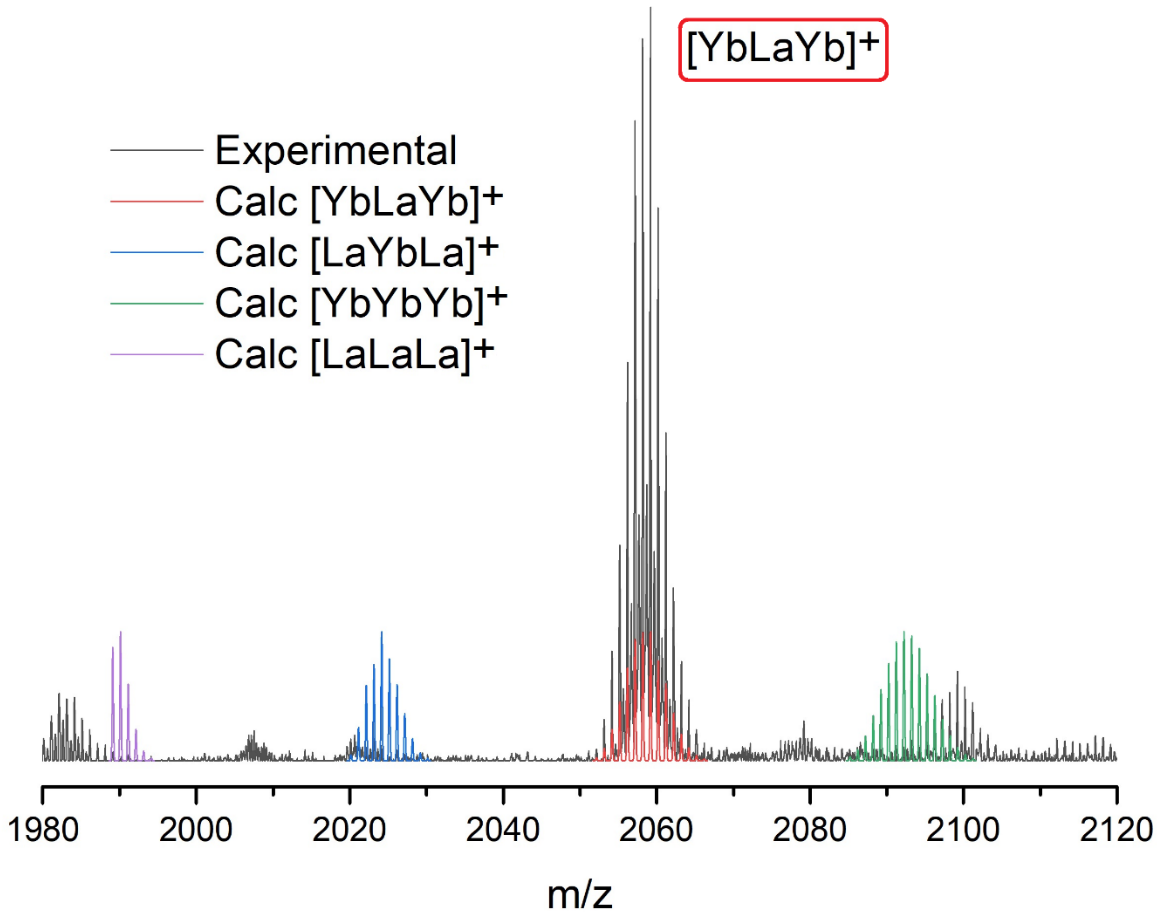

Figure S5. Selected region of the experimental (black line) ESI-MS spectrogram of compound **2** ([YbLaYb]), emphasizing the $[Yb_2La(LA)_2(LB)_2]^+$ (*m/z* = 2059.19) fragment, together with the corresponding calculated signals for the $[La_3]$ (purple line), $[YbLa_2]$ (blue line), $[Yb_2La]$ (red line) and $[Yb_3]$ (green line), metal distributions.

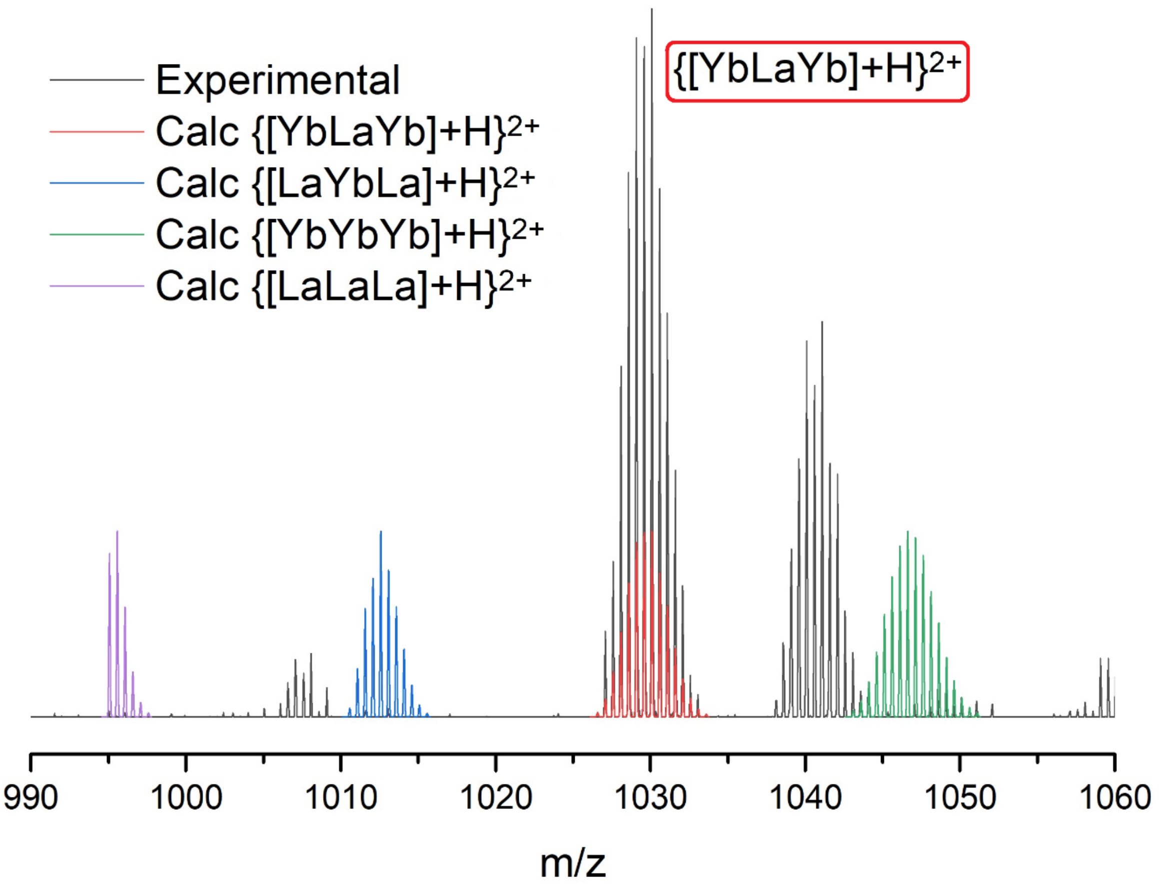

Figure S6. Selected region of the experimental (black line) ESI-MS spectrogram of compound **2** ([YbLaYb]), emphasizing the ($[Yb_2La(LA)_2(LB)_2]+H)^{2+}$ (*m/z* = 1030.09) fragment, together with the corresponding calculated signals for the $[La_3]$ (purple line), $[YbLa_2]$ (blue line), $[Yb_2La]$ (red line) and $[Yb_3]$ (green line), metal distributions.

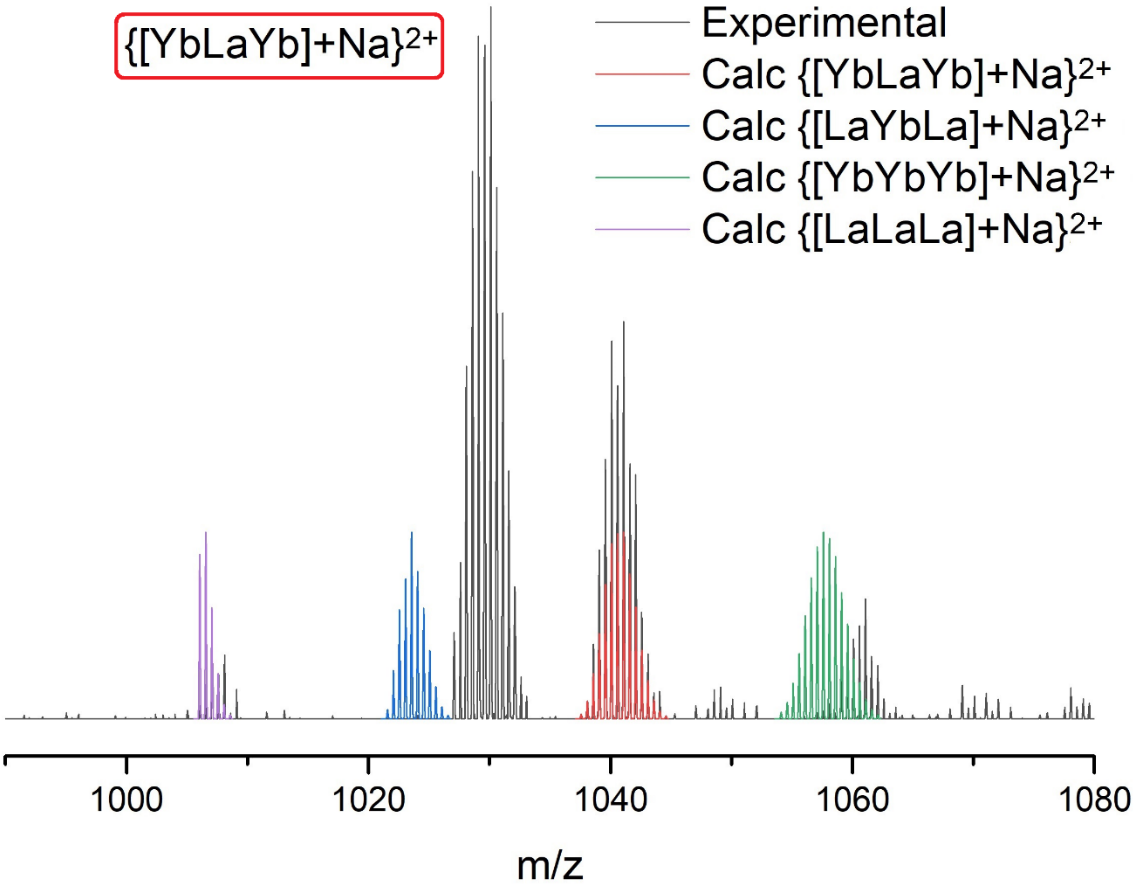

Figure S7. Selected region of the experimental (black line) ESI-MS spectrogram of compound **2** ([YbLaYb]), emphasizing the ($[Yb_2La(LA)_2(LB)_2]+Na)^{2+}$ (*m/z* = 1041.08) fragment, together with the corresponding calculated signals for the $[La_3]$ (purple line), $[YbLa_2]$ (blue line), $[Yb_2La]$ (red line) and $[Yb_3]$ (green line), metal distributions.

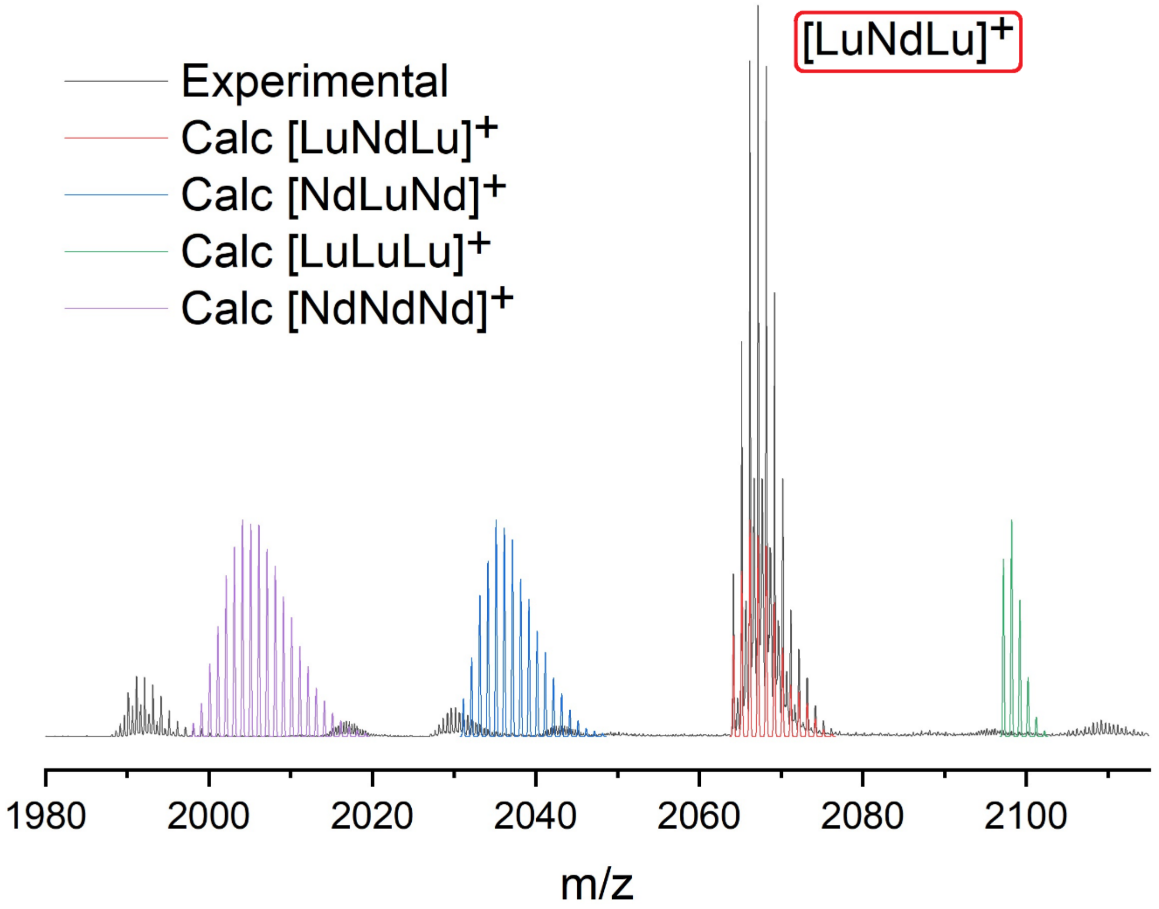

Figure S8. Selected region of the experimental (black line) ESI-MS spectrogram of compound **3** ([LuNdLu]), emphasizing the $[Lu_2Nd(LA)_2(LB)_2]^+$ (*m/z* = 2067.1647) fragment, together with the corresponding calculated signals for the $[Nd_3]$ (purple line), $[LuNd_2]$ (blue line), $[Lu_2Nd]$ (red line) and $[Lu_3]$ (green line), metal distributions.

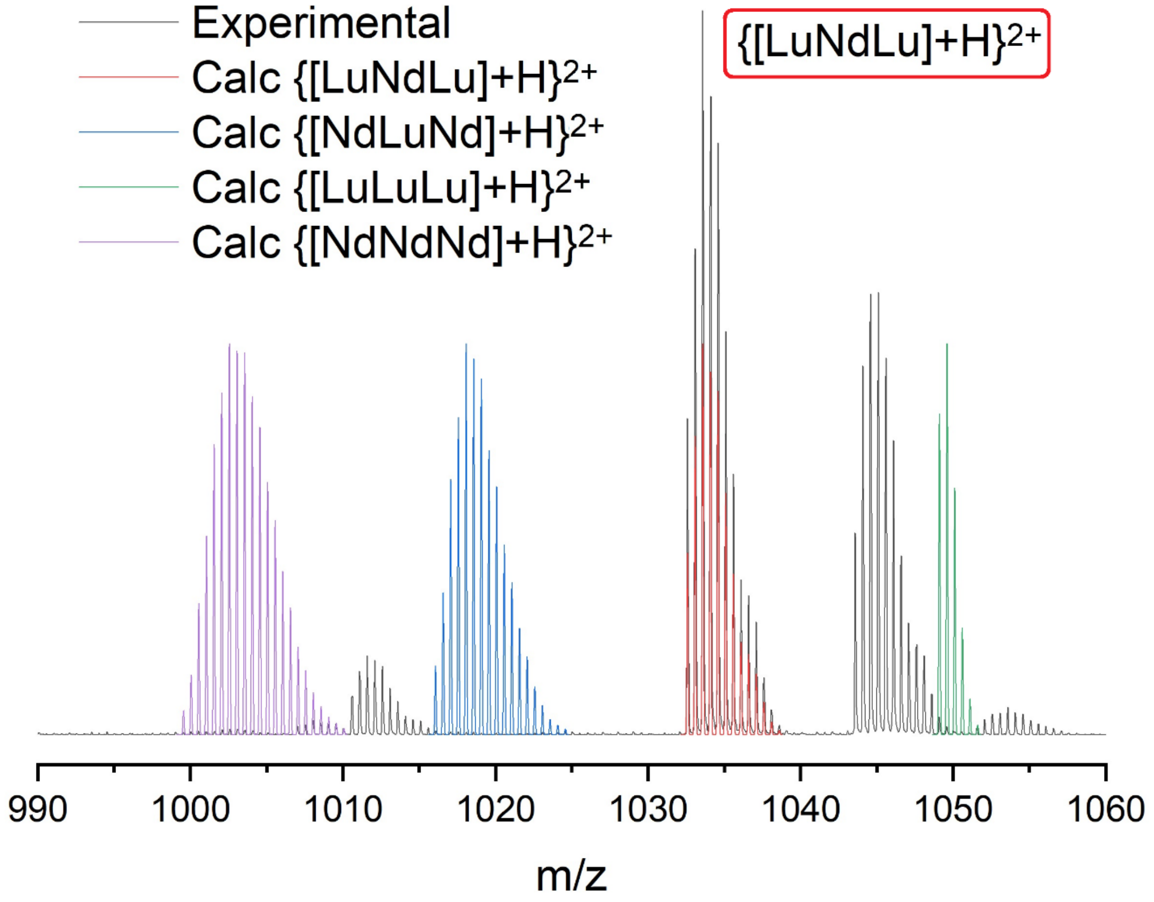

Figure S9. Selected region of the experimental (black line) ESI-MS spectrogram of compound **3** ([LuNdLu]), emphasizing the ([$Lu_2Nd(LA)_2(LB)_2$]+H)$^{2+}$ (*m/z* = 1033.5924) fragment, together with the corresponding calculated signals for the [Nd_3] (purple line), [$LuNd_2$] (blue line), [Lu_2Nd] (red line) and [Lu_3] (green line), metal distributions.

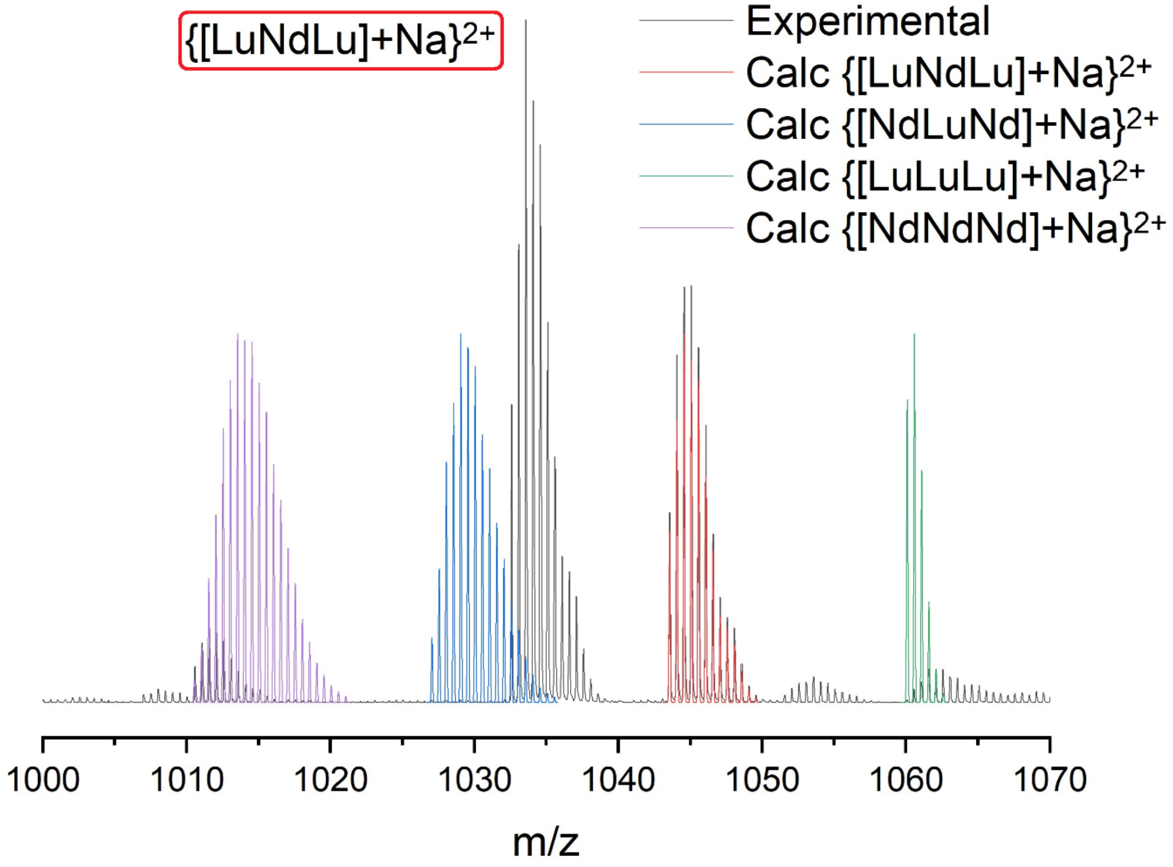

Figure S10. Selected region of the experimental (black line) ESI-MS spectra of compound **3** ([LuNdLu]), emphasizing the ($[Lu_2Nd(LA)_2(LB)_2]+Na)^{2+}$ (*m/z* = 1045.0863) fragment, together with the corresponding calculated signals for the [Nd_3] (purple line), [$LuNd_2$] (blue line), [Lu_2Nd] (red line) and [Lu_3] (green line), metal distributions.

Single-crystal X-ray diffraction

Data for compound **[NdYb$_2$(LA)$_2$(LB)$_2$(py)(H$_2$O)$_2$](NO$_3$) (1)** were obtained at 100 K with a Bruker APEX II QUAZAR diffractometer equipped with a microfocus multilayer monochromator with Mo Kα (λ = 0.71073 Å). Data for compound **[LaYb$_2$(LA)$_2$(LB)$_2$(py)(H$_2$O)$_2$](NO$_3$) (2)** were collected at 100 K at Beamline 12.2.1 of the Advanced Light Source (Berkeley, USA), on a Bruker D8 diffractometer equipped with a PHOTON II detector and using silicon (111) monochromated synchrotron radiation (λ = 0.7288 Å). Data for compound **[NdLu$_2$(LA)$_2$(LB)$_2$(py)(H$_2$O)$_2$](NO$_3$) (3)** were acquired at 100 K on the BL13-XALOC beamline[3] of the ALBA synchrotron (λ = 0.72932 Å). Data reduction and absorption corrections for **1** and **2** were performed with respectively SAINT and SADABS.[4] Data reduction for compound **3** were done with autoproc package[5] and XDS.[6] All structures were solved with Olex2[7] (**1**) or SHELXT[8] (**2** and **3**) and refined by full-matrix least-squares on F^2 with SHELXL.[9] In the structures of **2** and **3**, a portion of the lattice solvent molecules were too diffuse/disordered to be modelled satisfactorily. The corresponding void spaces were thus analysed and taken into account with PLATON/SQUEEZE[10] (**2**) or Olex2 (**3**), the formula reflecting the squeezed/masked content. The lanthanide sites composition in the model is supported by the worse agreement factors and unrealistic relative Ueq values observed for any other combination of the lanthanide sites.

All details can be found in CCDC 2209574-2209575-2209576 (**1**-**2**-**3**) which contain the supplementary crystallographic data for this paper. These data can be obtained free of charge from The Cambridge Crystallographic Data Center via https://summary.ccdc.cam.ac.uk/structure-summary-form. Crystallographic and refinement parameters are summarized in Table S1**,** while Tables S2 and S3 provide Ln–N bond lengths and details of hydrogen bonds in the structures of **1**, **2**, and **3**.

Table S1. Crystallographic and refinement parameters for the structure of compounds $[NdYb_2(LA)_2(LB)_2(py)(H_2O)_2](NO_3)\cdot 11py$ (**1**), $[LaYb_2(LA)_2(LB)_2(py)(H_2O)_2](NO_3)\cdot 10py$ (**2**), and $[NdLu_2(LA)_2(LB)_2(py)(H_2O)_2](NO_3)\cdot 10py$ (**3**).

Compound	**1**	**2**	**3**
Formula	$C_{160}H_{124}N_{17}$ $NdO_{21}Yb_2$	$C_{155}H_{119}LaN_{16}$ $O_{21}Yb_2$	$C_{155}H_{119}Lu_2N_{16}$ NdO_{21}
FW (g mol^{-1})	3111.07	3026.65	3035.84
Wavelength (Å)	0.71073	0.7288	0.72932
T (K)	100(2)	100(2)	100(2)
Crystal system	triclinic	triclinic	triclinic
Space group	P–1	P–1	P–1
a (Å)	16.5486(13)	16.2940(9)	16.365(3)
b (Å)	19.5401(17)	19.4380(9)	19.417(4)
c (Å)	23.393(2)	23.3614(12)	23.312(5)
α (°)	108.574(4)	108.353(2)	108.48(3)
β (°)	109.018(4)	108.685(2)	108.66(3)
γ (°)	92.015(4)	91.825(3)	92.01(3)
V (Å^3)	6697.1(10)	6579.7(6)	6579(3)
Z	2	2	2
ρ_{calcd} (g cm^{-3})	1.543	1.528	1.533
μ (mm^{-1})	1.874	2.072	1.148
Reflections	15278	25351	16701
R_{int}	0.0371	0.0428	0.0798
Restraints	123	316	320
Parameters	1810	1625	1668
S	1.045	1.028	0.975
R_1 [I>2σ(I)]	0.0390	0.0747	0.0702
wR_2 [I>2σ(I)]	0.1090	0.1681	0.1826
R_1 [all data]	0.0439	0.1130	0.0899
wR_2 [all data]	0.1154	0.2038	0.1949
Largest peak / hole (e Å^3)	1.564 / –1.125	4.437 / –2.350	2.960 / –1.273

Table S2. Ln-N and Ln-O bond lengths and Ln⋯Ln intramolecular separations in the structures of compounds **1**, **2**, and **3**.

**1**		**2**		**3**	
Yb1–O1	2.319(4)	Yb1–O1	2.327(6)	Lu1–O1	2.336(6)
Yb1–O3	2.319(4)	Yb1–O3	2.320(6)	Lu1–O3	2.304(6)
Yb1–O5	2.282(4)	Yb1–O5	2.288(6)	Lu1–O5	2.245(7)
Yb1–O6	2.300(4)	Yb1–O6	2.310(6)	Lu1–O6	2.322(7)
Yb1–O9	2.245(4)	Yb1–O9	2.240(6)	Lu1–O9	2.281(7)
Yb1–O10	2.352(4)	Yb1–O10	2.362(6)	Lu1–O10	2.376(4)
Yb1–O17	2.305(4)	Yb1–O17	2.272(6)	Lu1–O18	2.298(7)
Yb1–N1	2.371(5)	Yb1–N1	2.383(8)	Lu1–N1	2.386(7)
Yb2–O7	2.381(4)	Yb2–O7	2.378(5)	Lu2–O7	2.362(5)
Yb2–O8	2.273(4)	Yb2–O8	2.283(6)	Lu2–O8	2.215(8)
Yb2–O11	2.305(4)	Yb2–O11	2.313(6)	Lu2–O11	2.298(7)
Yb2–O12	2.236(4)	Yb2–O12	2.229(6)	Lu2–O12	2.274(6)
Yb2–O13	2.328(4)	Yb2–O13	2.322(6)	Lu2–O13	2.350(6)
Yb2–O15	2.292(4)	Yb2–O15	2.294(6)	Lu2–O15	2.297(6)
Yb2–O18	2.324(4)	Yb2–O18	2.327(6)	Lu2–O17	2.289(6)
Yb2–N4	2.389(5)	Yb2–N4	2.382(8)	Lu2–N4	2.371(7)
Nd1–O3	2.629(4)	La1–O3	2.646(6)	Nd1–O3	2.692(5)
Nd1–O4	2.446(4)	La1–O4	2.492(6)	Nd1–O4	2.471(7)
Nd1–O6	2.576(4)	La1–O6	2.619(5)	Nd1–O6	2.579(6)
Nd1–O7	2.671(4)	La1–O7	2.703(6)	Nd1–O7	2.717(7)
Nd1–O10	2.716(4)	La1–O10	2.724(6)	Nd1–O10	2.683(7)
Nd1–O11	2.561(4)	La1–O11	2.614(5)	Nd1–O11	2.581(5)
Nd1–O15	2.691(4)	La1–O15	2.699(6)	Nd1–O15	2.637(6)
Nd1–O16	2.481(4)	La1–O16	2.526(7)	Nd1–O16	2.450(7)
Nd1–N2	2.749(4)	La1–N2	2.781(7)	Nd1–N2	2.770(8)
Nd1–N3	2.773(4)	La1–N3	2.799(8)	Nd1–N3	2.750(8)
Nd1–N5	2.742(5)	La1–N5	2.808(8)	Nd1–N5	2.747(7)
Yb1⋯Nd1	3.9294(5)	Yb1⋯La1	3.9424(6)	Lu1⋯Nd1	3.9337(17)
Yb2⋯Nd1	3.9313(5)	Yb2⋯La1	3.9454(6)	Lu2⋯Nd1	3.9325(18)
Yb1⋯Yb2	7.851(1)	Yb1⋯Yb2	7.880(1)	Lu1⋯Lu2	7.857(2)

Table S3. Hydrogen bonds in the structures of compounds **1**, **2**, and **3**.

	D–H···A	D–H (Å)	H···A (Å)	D···A (Å)	D–H···A (º)
**1**	O17–H17A···N2S	0.95	2.24	2.714(6)	109.4
	O17–H17B···N1S	0.95	1.92	2.733(6)	141.8
	O18–H18B···N3S	0.95	2.28	2.745(6)	109.6
	O18–H18A···O13#1	0.95	1.93	2.731(5)	141.0
**2**	O17–H17B···N1S	0.911(19)	1.81(2)	2.718(11)	171(10)
	O17–H17C···N3S	0.947(18)	1.80(3)	2.74(2)	168(7)
	O17–H17C···N8S	0.947(18)	1.73(3)	2.669(15)	171(7)
	O18–H18C···N2S#1	0.90(2)	1.88(3)	2.77(2)	166(7)
	O18–H18B···O1#1	0.89(2)	2.41(3)	2.73 (2)	101(7)
**3**	O17–H17B···N1S	0.90(2)	1.95(8)	2.703(13)	141(11)
	O17–H17C···N2S	0.88(2)	1.87(4)	2.726(10)	164(10)
	O18–H18C···N3S	0.93(2)	2.00(4)	2.767(14)	138(5)
	O18–H18D···O1#1	0.90(2)	1.95(4)	2.733(8)	145(6)

#1 = 1-x, 1-y, 2-z

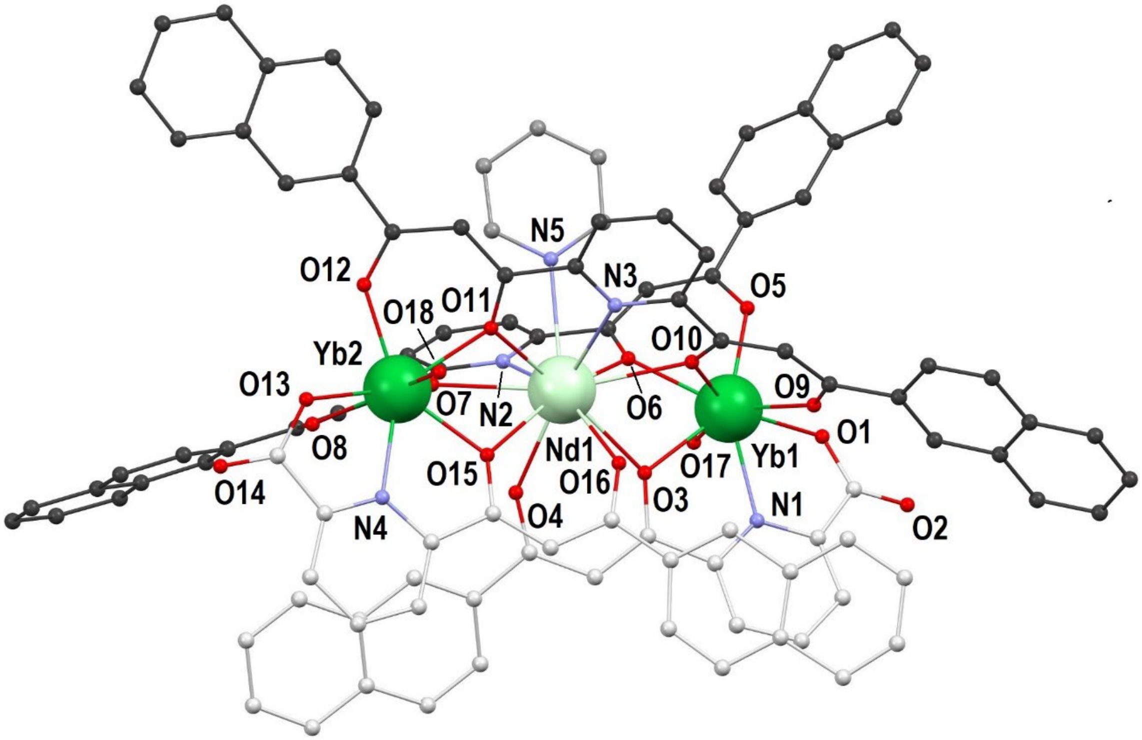

Figure S11. View of the cation $[Yb_2Nd(LA)_2(LB)_2(py)(H_2O)_2]^+$ of **1** with heteroatoms labelled (C atoms of LA^{2-}, py and LB^{2-} in dark, medium and light grey, respectively).

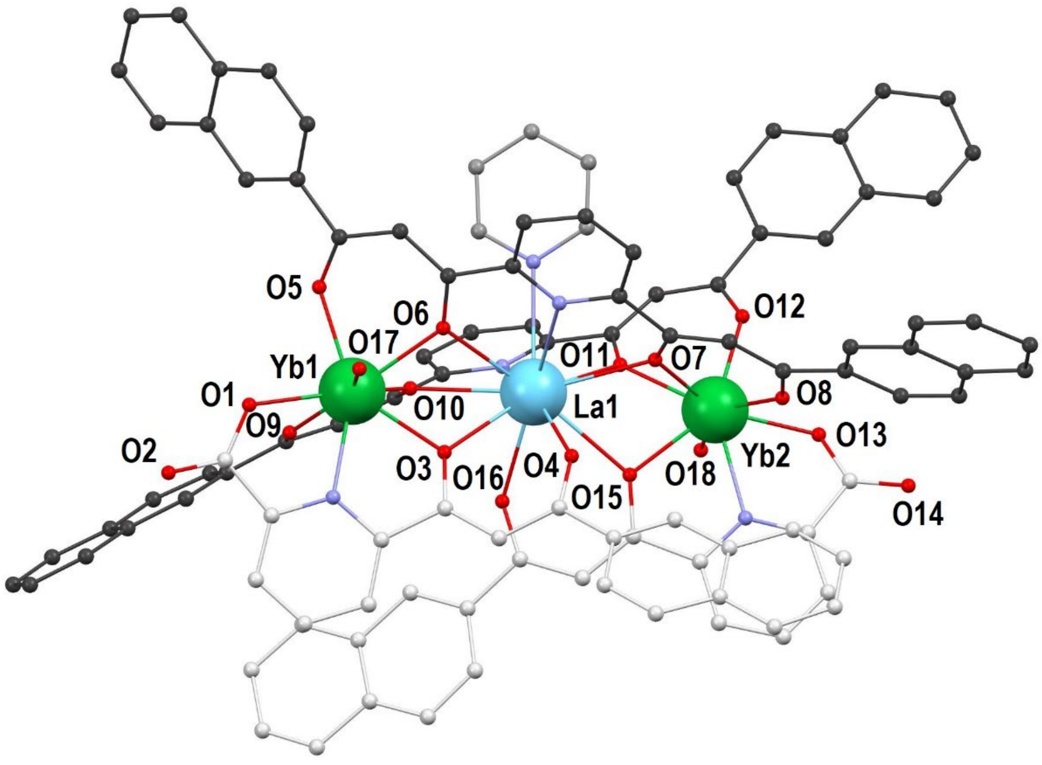

Figure S12. View of the cation $[Yb_2La(LA)_2(LB)_2(py)(H_2O)_2]^+$ of **2** with heteroatoms labelled (C atoms of LA^{2-}, py and LB^{2-} in dark, medium and light grey, respectively).

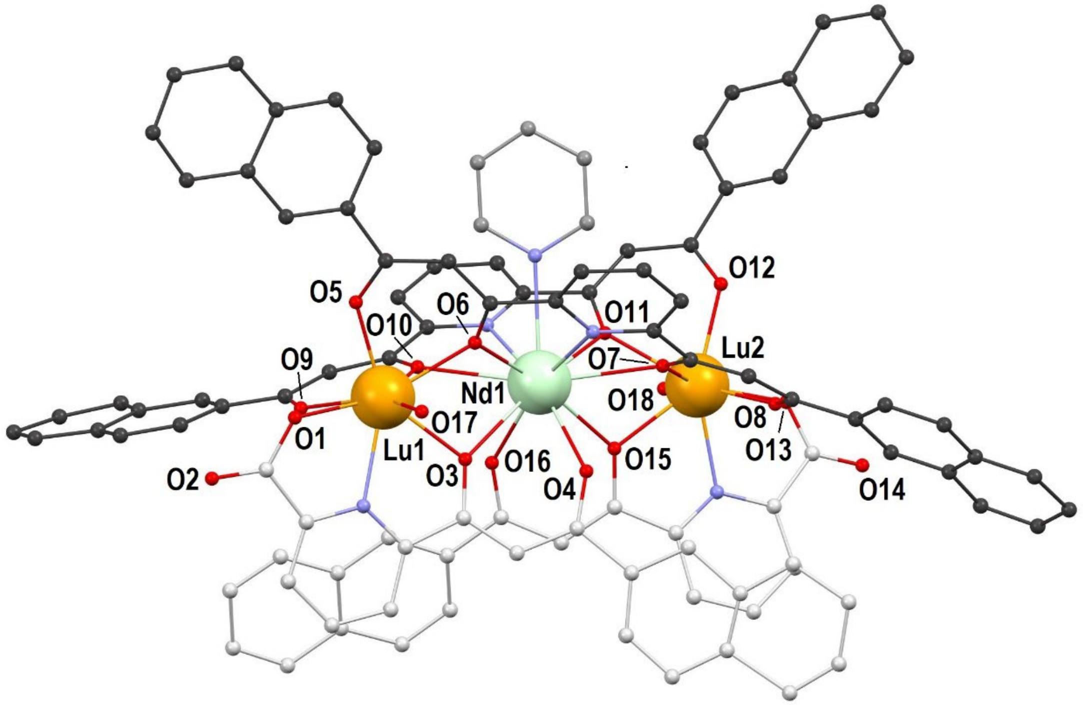

Figure S13. View of the cation $[Lu_2Nd(LA)_2(LB)_2(py)(H_2O)_2]^+$ of **3** with heteroatoms labelled (C atoms of LA^{2-}, py and LB^{2-} in in dark, medium and light grey, respectively).

Photo physical Studies

The luminescence spectra were measured using a Horiba-Jobin Yvon Fluorolog-3® spectrofluorimeter, equipped with a three slit double grating excitation monochromator with dispersions of 2.1 nm/mm (1200 grooves/mm) and a single grating iHR320 monochromator for the emission with a dispersion of 20 nm/mm (150 grooves/mm). The steady-state luminescence was excited by unpolarized light from a 450 W xenon CW lamp and detected at an angle of 90° through a FGL850 filter by a Symphony® II CCD detector. Spectra were reference corrected for both the excitation source light intensity variation (lamp and grating) and the emission spectral response (detector and grating).

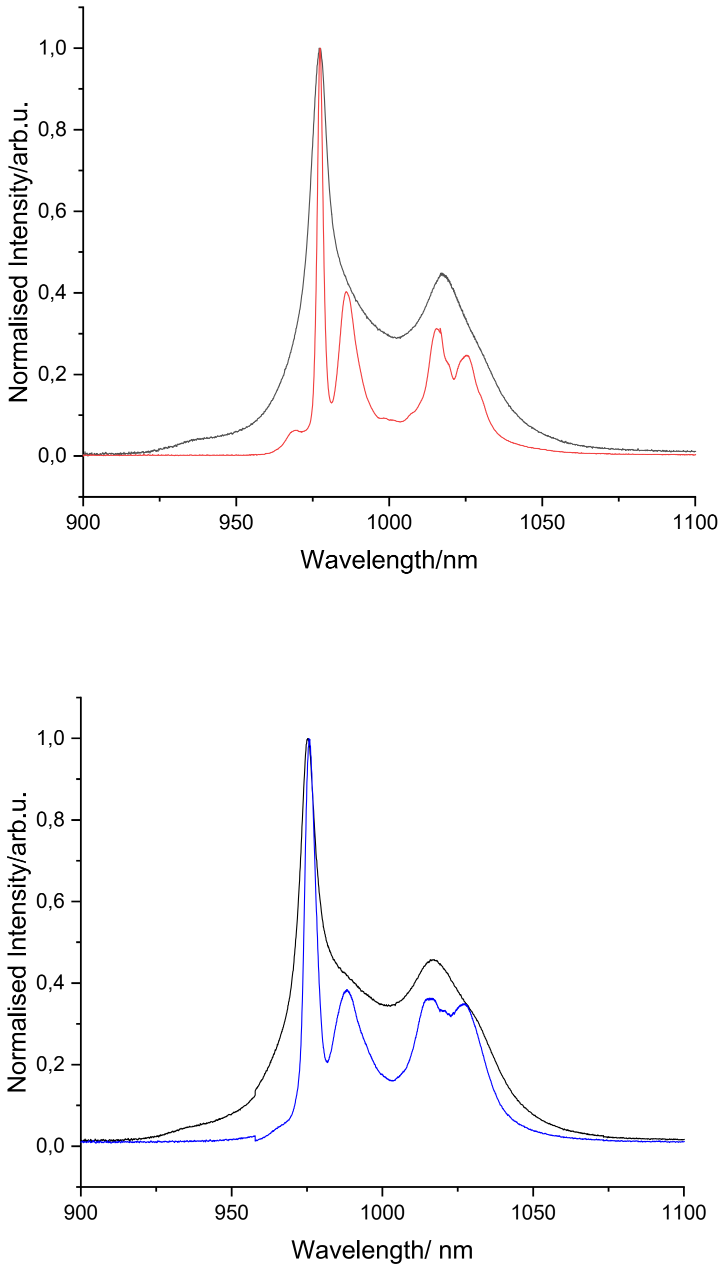

Figure S14. Normalised emission (λ_{exc}=400 nm) of [YbLaYb] (**2**) in (top) diluted MeOH:DMSO solution (1:1) and (bottom) the solid state at room temperature (black trace) and 77K (red and blue traces, respectively).

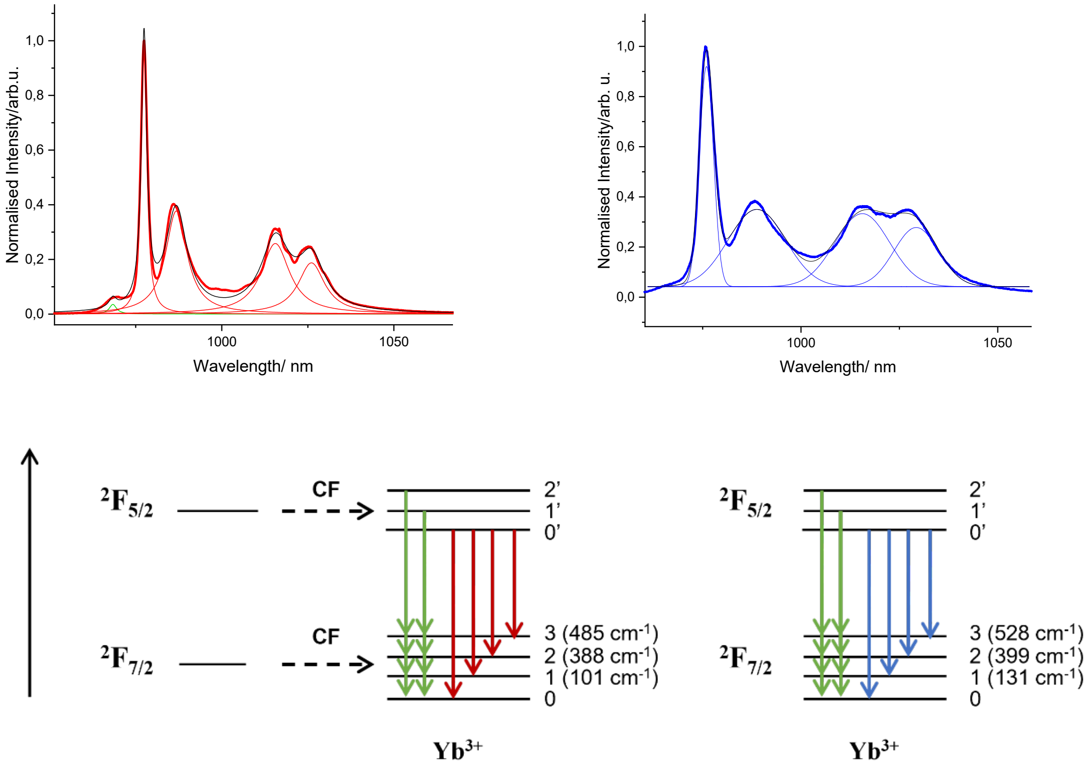

Figure S15. Deconvolution of the emission spectra plots of complex [YbLaYb] (**2**) and the correspondent assignment of energy to the Yb^{3+} Kramer states, in frozen MeOH:DMSO solution (1:1), (left, red trace) and solid state at 77K (right, blue trace).

Table S4. Data obtained from the deconvoluted emission spectra of complex [YbLaYb] (**2**) in frozen MeOH:DMSO solution (1:1).

Model	Lorentz				
Equation	$y = y_0 + (2A/\pi)(w/(4(x-xc)^2 + w^2))$				
Plot	Peak1(Normalized Y1)	Peak2(Normalized Y1)	Peak3(Normalized Y1)	Peak4(Normalized Y1)	Peak5(Normalized Y1)
y0	2,56529E-4 ± 3,31421E-4	2,56529E-4 ± 3,31421E-4	2,56529E-4 ± 3,31421E-4	2,56529E-4 ± 3,31421E-4	2,56529E-4 ± 3,31421E-4
xc	968,3682 ± 0,14208	977,4176 ± 0,00439	986,7868 ± 0,02336	1015,56876 ± 0,05537	1026,05592 ± 0,07218
w	2,57749 ± 0,41528	1,93068 ± 0,01359	7,53464 ± 0,07604	10,05175 ± 0,17331	9,4164 ± 0,2269
A	0,14236 ± 0,01663	2,99396 ± 0,0162	4,4596 ± 0,03425	4,07538 ± 0,07637	2,76982 ± 0,07365
Reduced Chi-Sqr	1,32268E-4				
R-Square (COD)	0,99047				
Adj. R-Square	0,9904				

Table S5. Data obtained from the deconvoluted emission spectra of complex [YbLaYb] (**2**) in the solid state at 77K.

Model	Gauss			
Equation	$y=y_0 + (A/(w\sqrt{(\pi/2)}))*\exp(-2((x-xc)/w^2)$			
Plot	Peak1(Normalized Y2)	Peak2(Normalized Y2)	Peak3(Normalized Y2)	Peak4(Normalized Y2)
y0	0,04205 ± 0,00117	0,04205 ± 0,00117	0,04205 ± 0,00117	0,04205 ± 0,00117
xc	975,97381 ± 0,00933	988,7634 ± 0,06775	1015,58816 ± 0,30665	1029,28452 ± 0,29008
w	3,56352 ± 0,02219	14,5632 ± 0,20645	13,86726 ± 0,45455	11,31544 ± 0,32786
A	3,92021 ± 0,02953	5,61944 ± 0,07436	5,05912 ± 0,22727	3,3431 ± 0,22138
Reduced Chi-Sqr	2,63399E-4			
R-Square (COD)	0,99167			
Adj. R-Square	0,99155			

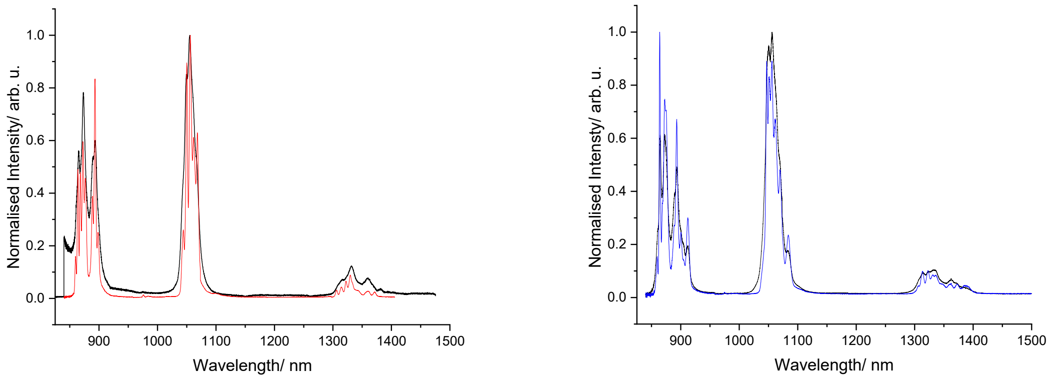

Figure S16. Normalised emission (λ_{exc}=400 nm) of [LuNdLu] (**3**) in (left) diluted MeOH:DMSO solution (1:1) and (right) the solid state at room temperature (black trace) and 77K (red and blue traces, respectively).

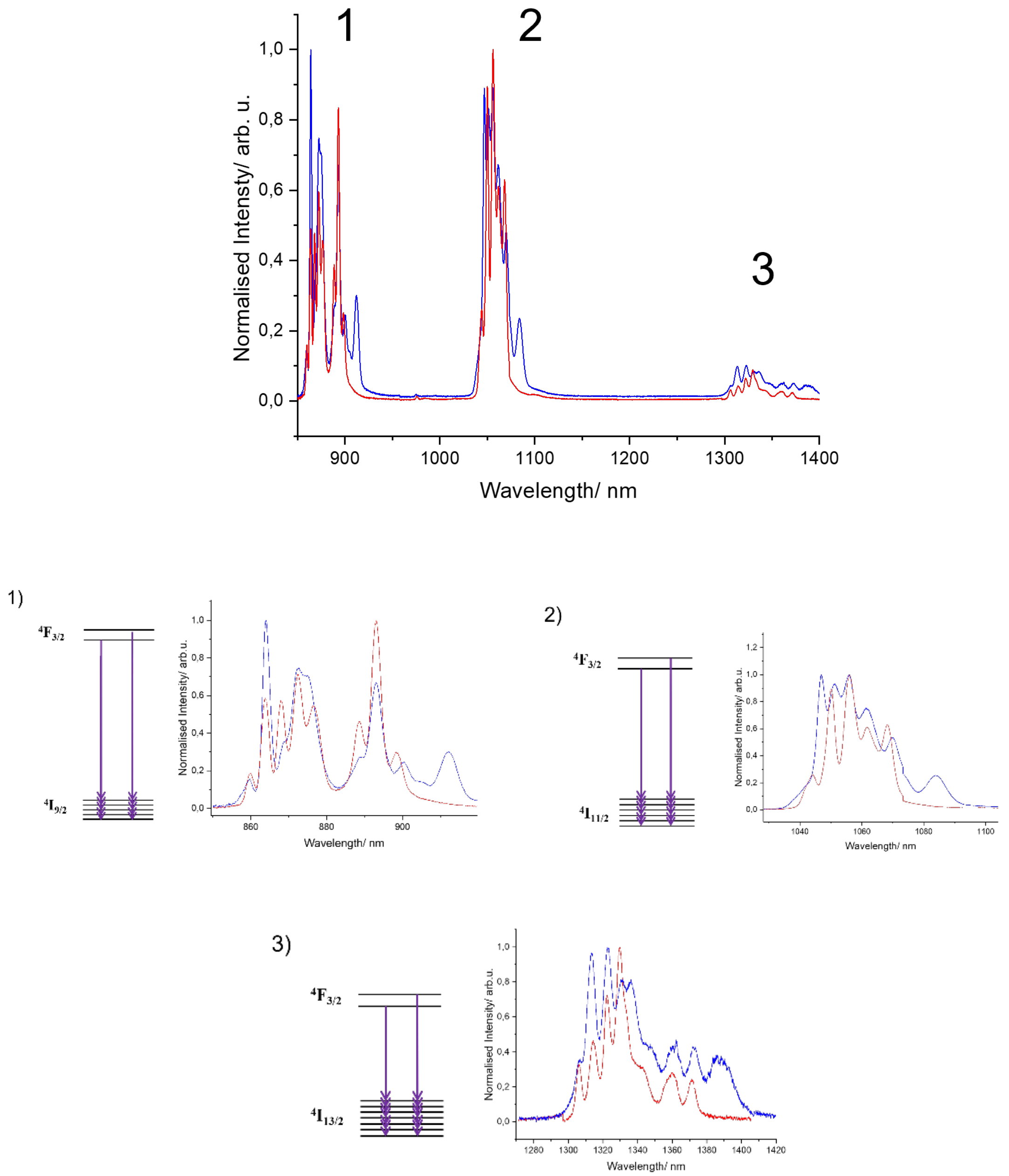

Figure S17. Normalised emission (λ_{exc}=400 nm) of of [LuNdLu] **3** in frozen solution (red trace) and in the solid state at 77K (blue trace) with their expanded regions of emission corresponding to the $^4F_{3/2} \rightarrow {}^4I_{9/2}$, $^4F_{3/2} \rightarrow {}^4I_{11/2}$ and $^4F_{3/2} \rightarrow {}^4I_{13/2}$ transitions, respectively.

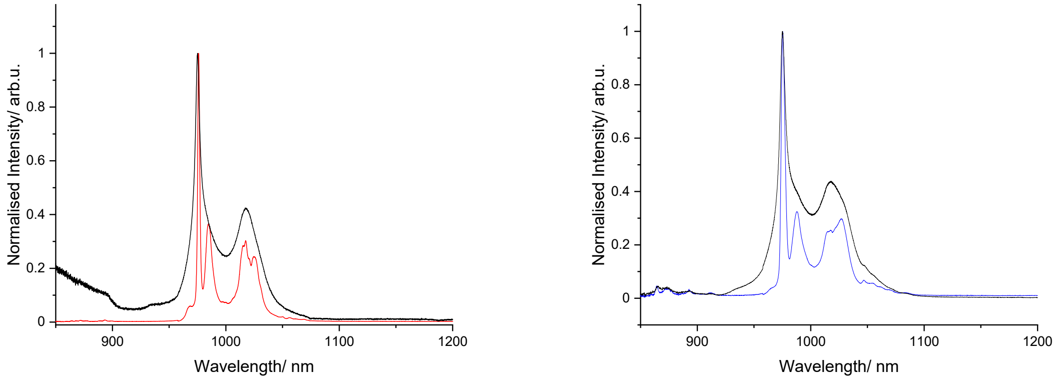

Figure S18. Normalised emission (λ_{exc}=400 nm) of [YbNdYb] (**1**) in (left) diluted MeOH:DMSO solution (1:1) and (right) the solid state at room temperature (black trace) and 77K (red and blue traces, respectively).

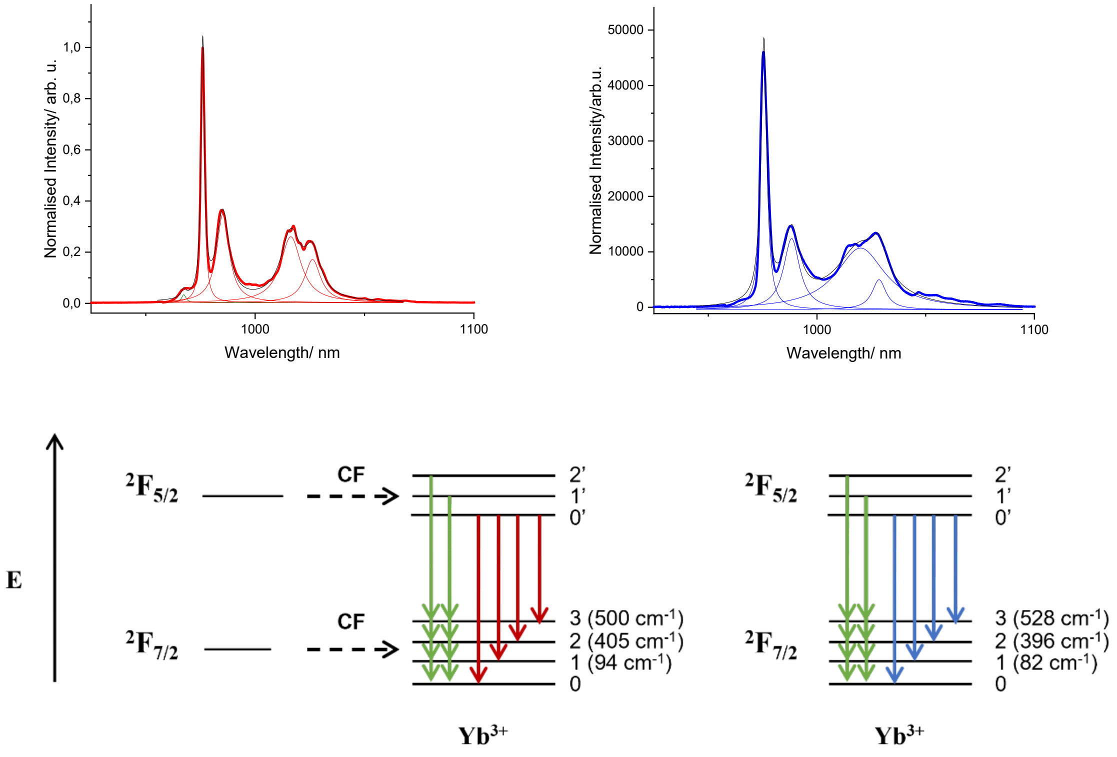

Figure S19. Deconvolution of the emission spectra plots of of [YbNdYb] (**1**) and the correspondent assignment of energy to the Yb^{3+} Kramer states, in frozen solution (red trace) and solid state at 77K (blue trace).

Table S6. Data obtained from the deconvoluted emission spectra of complex [YbNdYb] (**1**) in frozen MeOH:DMSO solution (1:1).

Model	Lorentz				
Equation	$y = y_0 + (2A/\pi)(w/(4(x-xc)^2 + w^2))$				
Plot	Peak1(B)	Peak2(B)	Peak3(B)	Peak4(B)	Peak5(B)
y0	0,00267 ± 8,31566E-4	0,00267 ± 8,31566E-4	0,00267 ± 8,31566E-4	0,00267 ± 8,31566E-4	0,00267 ± 8,31566E-4
xc	967,38956 ± 0,18092	976,05934 ± 0,00486	985,25356 ± 0,02775	1016,30908 ± 0,07908	1026,26146 ± 0,09199
w	2,48921 ± 0,54412	1,8354 ± 0,01496	6,93464 ± 0,09225	11,41685 ± 0,23298	8,85459 ± 0,30089
A	0,12155 ± 0,02003	2,86756 ± 0,01787	3,77024 ± 0,04038	4,62012 ± 0,10919	2,34565 ± 0,09604
Reduced Chi-Sqr	1,7051E-4				
R-Square (COD)	0,9913				
Adj. R-Square	0,99116				

Table S7. Data obtained from the deconvoluted emission spectra of complex [YbNdYb] (**1**) in the solid state at 77K.

Model	Lorentz			
Equation	$y = y_0 + (2A/\pi)(w/(4(x-xc)^2 + w^2))$			
Plot	Peak1(D)	Peak2(D)	Peak3(D)	Peak4(D)
y0	-420,99285 ± 63,20364	-420,99285 ± 63,20364	-420,99285 ± 63,20364	-420,99285 ± 63,20364
xc	975,59014 ± 0,01078	988,3552 ± 0,0676	1020,04435 ± 0,3769	1028,55961 ± 0,17207
w	3,25197 ± 0,03375	9,03513 ± 0,23434	27,40991 ± 0,63211	7,72344 ± 0,92615
A	238282,02653 ± 1928,31812	181740,07602 ± 3862,74546	478048,44129 ± 19085,23064	65434,46213 ± 11387,61628
Reduced Chi-Sqr	1031867,82859			
R-Square (COD)	0,97919			
Adj. R-Square	0,979			

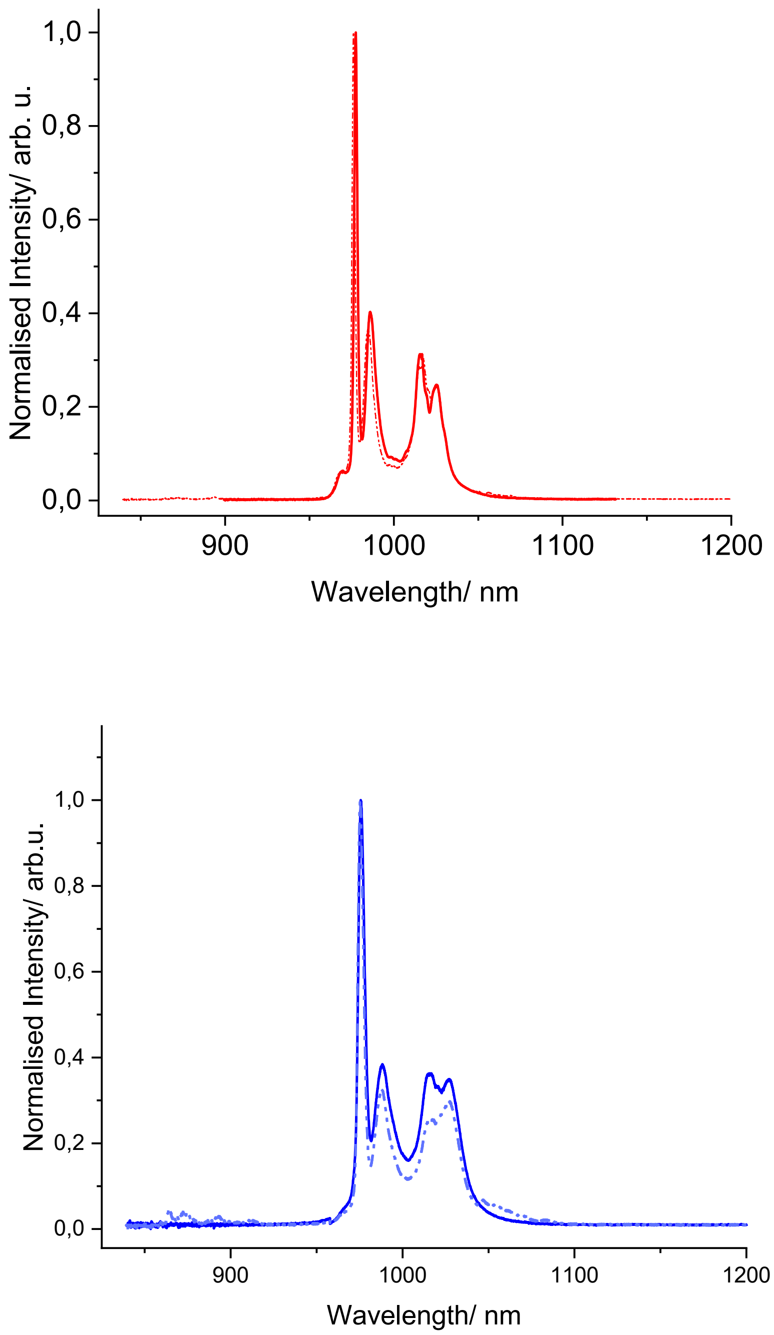

Figure S20. Comparison of the Yb^{3+} emission of complex **2** (full trace) and [YbNdYb] **1** (dotted line) in frozen solution (top, red trace) and solid state at 77K (bottom, blue trace).

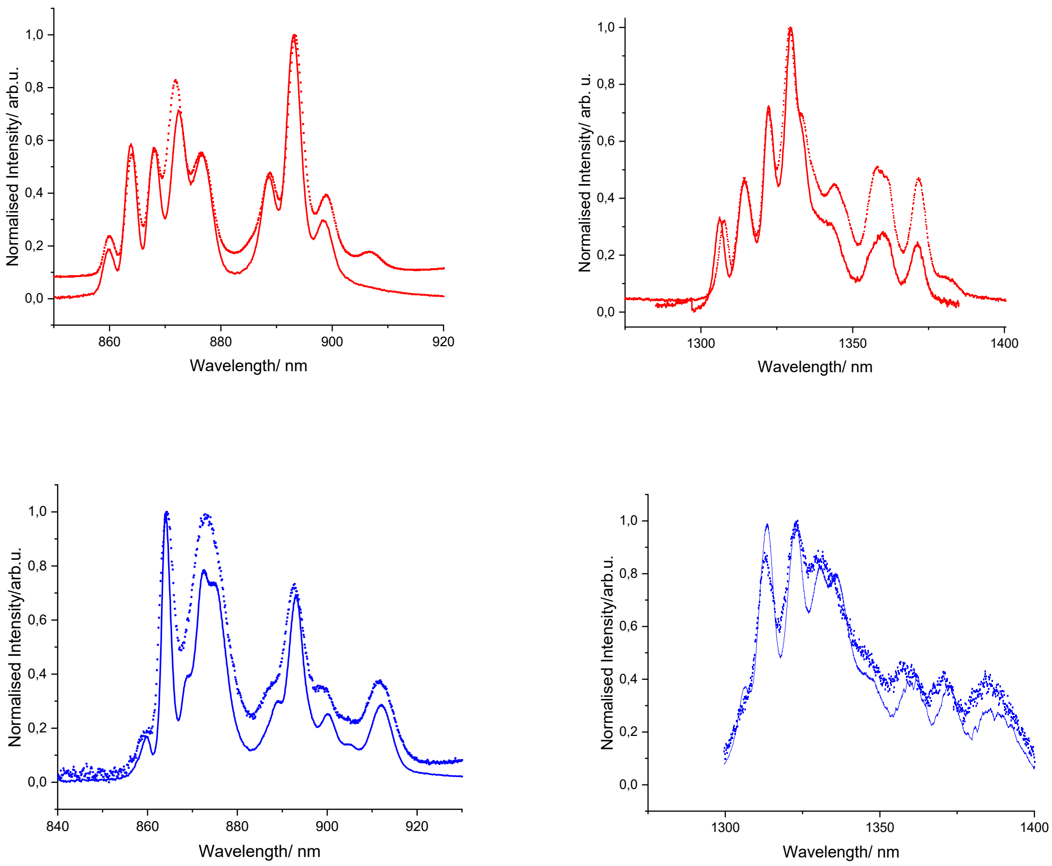

Figure S21. Comparison of the Nd^{3+} emission of [LuNdLu] **3** (full trace) and [YbNdYb] **1** (dotted line) in frozen solution (red trace) and solid state at 77K (blue trace).

---

[1] Barrios L. A., Peyrecave-Lleixa E., Craig G. A., Roubeau O., Teat S. J., Aromí G., *Eur. J. Inorg. Chem.*, **2014**, 6013-6021.

[2] Velasco V., Barrios L. A., Schütze M., Roubeau O., Luis F., Teat S. J., Aguilà D., Aromí G., *Chem., Eur. J.*, **2019**, *25*, 15228 – 15232.

[3] J. Juanhuix, F. Gil-Ortiz, G. Cuní, C. Colldelram, J. Nicolás, J. Lidón, E. Boter, C. Ruget, S. Ferrer and J. Benach, *J. Synchrotron Radiat.*, **2014**, *21*, 679-689

[4] a) G. M. Sheldrick, *SAINT and SADABS*, 2012, Bruker AXS Inc., Madison, Wisconsin, USA; b) L. Krause, R. Herbst-Irmer, G. M. Sheldrick, D. Stalke, *J. Appl. Cryst.*, **2015**, *48*, 3-10

[5] C. Vonrhein, C. Flensburg, P. Keller, A. Sharff, O. Smart, W. Paciorek, T. Womack, and G. Bricogne, *Acta Cryst. D,* **2011**, *67*, 293-302

[6] W. Kabsch, *Acta Cryst. D* **2010**, *66*, 125-132.

[7] L. J. Bourhis, O. V. Dolomanov, R. J. Gildea, J. A. K. Howard, H. Puschmann, *Acta Cryst. A* **2015**, *71*, 59-75.

[8] G. M. Sheldrick, *Acta Cryst. A* **2015**, *71*, 3-8.

[9] G. M. Sheldrick, *Acta Cryst. C* **2015**, *71*, 3-8.
[10] A. L. Spek, *Acta Cryst. C* **2015**, *71*, 9-18.
